# [^68^Ga]Ga-Ornibactin for *Burkholderia cepacia* complex Infection Imaging Using Positron Emission Tomography

**DOI:** 10.1021/acs.jmedchem.3c00469

**Published:** 2023-05-30

**Authors:** Katerina Bendova, Vladislav Raclavsky, Radko Novotny, Dominika Luptakova, Miroslav Popper, Zbynek Novy, Marian Hajduch, Milos Petrik

**Affiliations:** † Institute of Molecular and Translational Medicine, Faculty of Medicine and Dentistry and Czech Advanced Technology and Research Institute, Palacky University, Olomouc 779 00, Czech Republic; ‡ Department of Microbiology, Faculty of Medicine and Dentistry, Palacky University and University Hospital, Olomouc 775 15, Czech Republic; § Institute of Microbiology of the Czech Academy of Sciences, Laboratory of Molecular Structure Characterization, Prague 4 142 20, Czech Republic; ∥ Laboratory of Experimental Medicine, University Hospital, Olomouc 779 00, Czech Republic

## Abstract

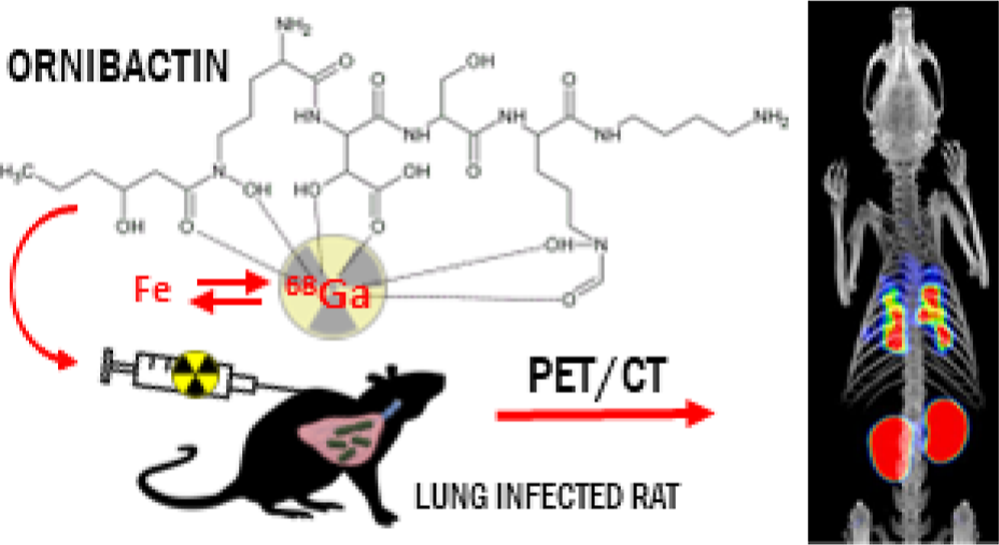

Bacteria from the *Burkholderia cepacia* complex are generally considered to be non-pathogenic to the healthy population. However, some of these species may cause serious nosocomial infections in immunocompromised patients; as such, it is essential to diagnose these infections rapidly so that adequate treatment can be initiated. We report here the use of a radiolabeled siderophore, ornibactin (ORNB), for positron emission tomography imaging. We successfully radiolabeled ORNB with gallium-68 with high radiochemical purity and proved that the resulting complex has optimal *in vitro* characteristics. In mice, the complex did not show excessive accumulation in organs and was excreted in the urine. We demonstrated that the [^68^Ga]Ga-ORNB complex accumulates at the site of *Burkholderia multivorans* infection, including pneumonia, in two animal infection models. These results suggest that [^68^Ga]Ga-ORNB is a promising tool for the diagnosis, monitoring, and evaluation of the therapeutic response to *B. cepacia* complex infection.

## Introduction

Pulmonary infections are the third most common cause of death worldwide.^1^ In healthcare settings, the situation is even more serious, as hospital-acquired pneumonia is the most frequently reported cause of nosocomial infections.^1^ These infections represent a serious problem, especially for immunocompromised patients as they can significantly increase mortality.^2,3^ Given the growing threat of multidrug-resistant microorganisms, particularly in the hospital setting, rapid and accurate identification of the causative organism is more urgent than ever so that infected patients can receive adequate and effective treatment. However, current diagnostic methods often lack specificity and sensitivity and may be too invasive or time-consuming for critically ill patients. These shortcomings mean that there is a high demand for the development of modern diagnostic tools.^2^

Although iron is the fourth most abundant element, its availability to microorganisms is very limited. In aerobic environments, iron occurs in the form of Fe^3+^, which is poorly soluble in water and, therefore, unavailable to living organisms.^4^ Bacteria have evolved different strategies to obtain this vital element, which is essential for their survival. A common tactic is bacterial synthesis and utilization of siderophores,^5^ which are low-molecular-weight iron chelators that are used for scavenging iron from the environment. These compounds are utilized not only by bacteria but also by various fungi and plants.^6^ Siderophores play an important role in essential microbial metabolism, pathogenicity, virulence, and–in some cases–biofilm formation.^5,7^ Bacteria produce siderophores under iron-limited conditions, *e.g.*, during infectious processes that involve host–bacterial competition for iron. Siderophores demonstrate significantly higher affinity for the ferric ion; as a result, the siderophores produced by bacteria remove iron from various iron-binding molecules in the host.^7^ In Gram-negative bacteria, the iron–siderophore complex is transported into the bacterial cytoplasm *via* an energy-dependent outer membrane receptor. Once within the cytoplasm, the iron is released from the binding site either by non-specific reductases or specific enzymes.^8^ Most bacteria obtain iron by producing their own siderophores, but they may also utilize so-called xenosiderophores, which are siderophores produced by other microorganisms (siderophore piracy).^9^

The *Burkholderia cepacia* complex (BCC) is a group of Gram-negative, obligately aerobic bacteria consisting of more than 22 known genetically distinct microbes.^10^ Although members of this complex are commonly found in the environment, are often used in agriculture, and are generally considered to be non-pathogenic to the healthy population, some species can cause serious healthcare-associated infections.^11^ These include surgical wound infections, urinary tract infections, septicemia, and pneumonia.^12^ Hospital-acquired pneumonia occur most commonly in immunocompromised patients, particularly those with granulomatous disease and cystic fibrosis (CF).^13,14^ Even though almost all BCC species have been isolated from the sputum of CF patients, 70% of the infections are caused by *Burkholderia multivorans* and *Burkholderia cenocepacia*.^15^*Burkholderia* colonization of the respiratory tract in these patients can be quite unpredictable and is generally associated with poor prognosis.^13^ It can either cause asymptomatic chronic infection or further deteriorate inflammation and lung function and–in some cases–develop into cepacia syndrome.^9^ This syndrome consists of necrotizing pneumonia, fever, rapid decline in respiratory function, and bacteremia, which significantly increases the mortality of infected patients and has been previously described in non-CF patients.^16,17^ BCC is even more threatening due to its resistance to various antibiotics and disinfectants, which enables BCC to spread through the hospital environment *via* contaminated equipment and medical solutions.^12,18,19^ For these reasons, no effective treatment strategies are currently available to eliminate this complex in CF patients.^20^ However, not only the therapy but also the diagnosis of BCC remains challenging and affects the prognosis of patients. It takes 48–72 h to cultivate BCC on selective soils with potential for false-negative results.^21^ Commercially available kits and automated systems for microbial identification often misidentify BCC bacteria.^22^ This makes molecular techniques, including polymerase chain reaction (PCR) and multi-locus sequence typing, the gold standard for BCC detection. However, these molecular techniques also have limitations. For instance, they may detect nucleic acids of an inactive pathogen or cannot provide adequate information to reliably identify the location of BCC infection. BCC bacteria synthesize four different types of siderophores: pyochelin, cepabactin, cepaciachelin, and ornibactin (ORNB).^9^ According to the bioinformatic analysis of the BCC genome, almost all bacteria in this complex are able to produce ORNB, while the other siderophores are only produced by specific members.^9^ ORNB occurs in three variants, which differ in acyl chain length and are referred to as ORNB-C4, -C6, and -C8.^23^ The uptake of Fe-ORNB depends on a specific outer membrane receptor.^24,25^ ORNB has previously been shown to play a crucial role in CF pathogenesis, as bacterial mutants with impaired ORNB uptake were less pathogenic compared to parental strains in murine models.^25^

**Figure 1:**
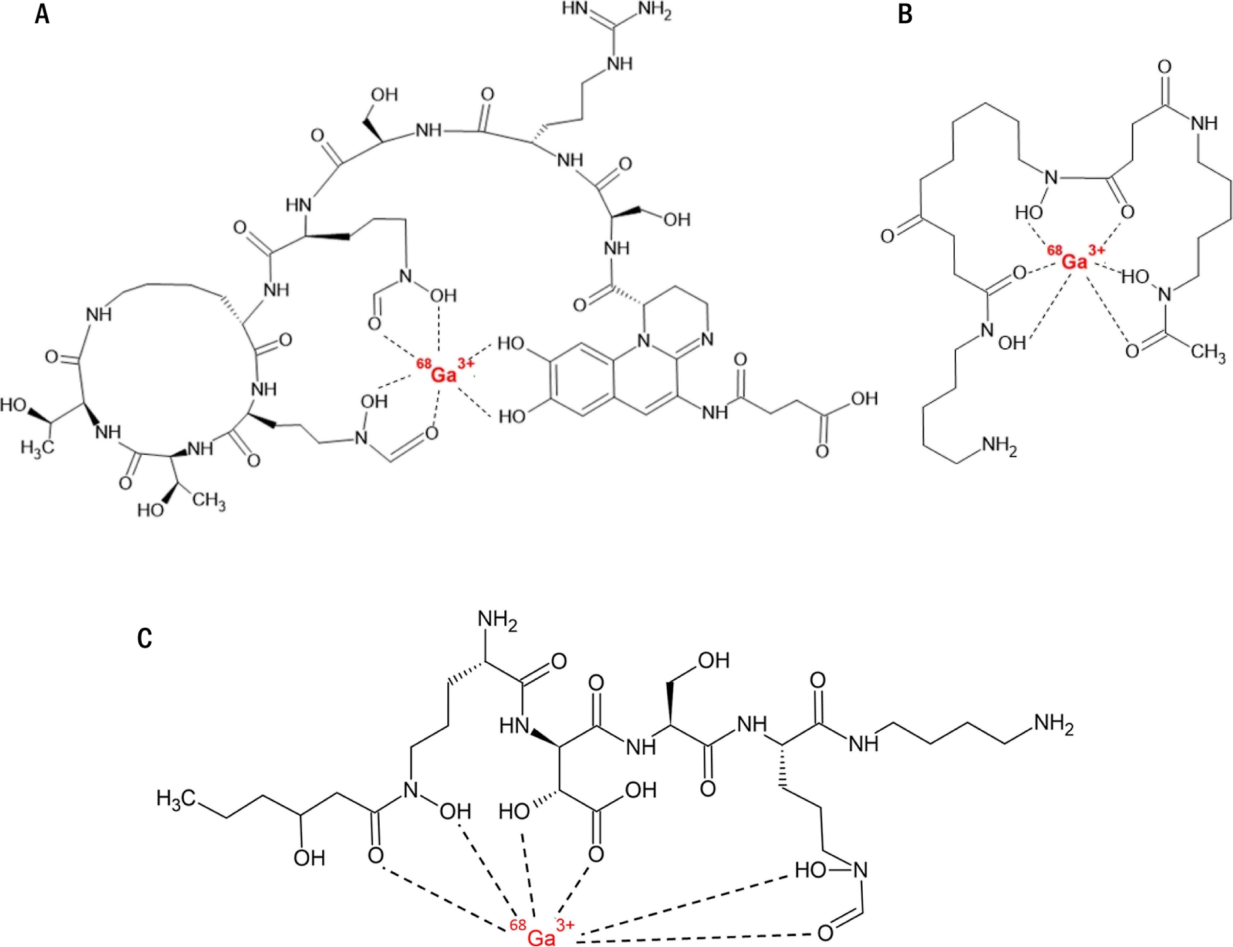
Examples of previously tested siderophores successfully used for PET imaging of bacteria from the literature: (A) [^68^Ga]Ga-Pyoverdine PAO1 and (B) [^68^Ga]Ga-Desferrioxamine-B.^28,29^ (C) The chemical structure of [^68^Ga]Ga-Ornibactin-C6.

In this study, we describe how gallium-68-labeled ORNB-C6 can be used for specific imaging of BCC infections *via* positron emission tomography (PET). Due to the similar physicochemical properties of iron and gallium, various siderophores can bind to gallium-68.^26^ Previous studies have shown that ^68^Ga-siderophores are actively taken up by a variety of microorganisms, which enables the imaging of microbial infection *via* PET (Figure 1A,B).^27−30^ Here, we aim to provide a new and promising diagnostic tool for the efficient detection of BCC members.

## Results

### Radiolabeling, Quality Control, and *In Vitro* Characterization of [^68^Ga]Ga-ORNB

ORNB was radiolabeled with gallium-68 with high radiochemical purity (>95%); this was confirmed both by reversed-phase radio high performance liquid chromatography (RP-radioHPLC) and radio instant thin layer chromatography (radio-iTLC) (Figure S1). The [^68^Ga]Ga-ORNB complex was highly stable in solution at a pH of 6 (>98%) and in human serum (>97%) after 2 h of incubation. In the solution with excess diethylenetriaminepentaacetic acid (DTPA) (6 mM), the stability of the [^68^Ga]Ga-ORNB complex decreased over time (<40% after 2 h). Moreover, [^68^Ga]Ga-ORNB rapidly degraded in a 0.1 M FeCl_3_ solution (<1% remained after 30 min). The complex demonstrated hydrophilic properties (log *P* = −2.65% ± 0.05) and low plasma protein binding (6.72% ± 0.71) after 2 h of incubation. The *in vitro* characteristics of [^68^Ga]Ga-ORNB are summarized in Table 1.

**Table 1:** *In Vitro* Characterization Results of [^68^Ga]Ga-ORNB^a^

log *P* (%)		protein binding (%)	stability in solution at pH = 6 (%)	stability in human serum (%)	stability in DTPA solution (%)	stability in iron solution (%)
(*n* = 6)	incubation time (min)	(*n* = 3)	(*n* = 3)	(*n* = 3)	(*n* = 3)	(*n* = 3)
–2.67 ± 0.05	30	4.51 ± 1.88	98.39 ± 0.55	97.65 ± 1.10	80.43 ± 3.86	0.90 ± 0.20
	60	5.26 ± 0.39	98.90 ± 0.88	98.50 ± 0.53	70.87 ± 4.07	0.40 ± 0.36
	120	6.72 ± 0.71	99.11 ± 0.42	97.99 ± 1.35	60.80 ± 4.71	0.27 ± 0.06

a Log *P*, protein binding, and stability in solution results at pH = 6, in human serum, in solution with excess DTPA, and in solution with excess iron.

### *In Vitro* Uptake Assays of [^68^Ga]Ga-ORNB in Microbial Cultures

**Figure 2:**
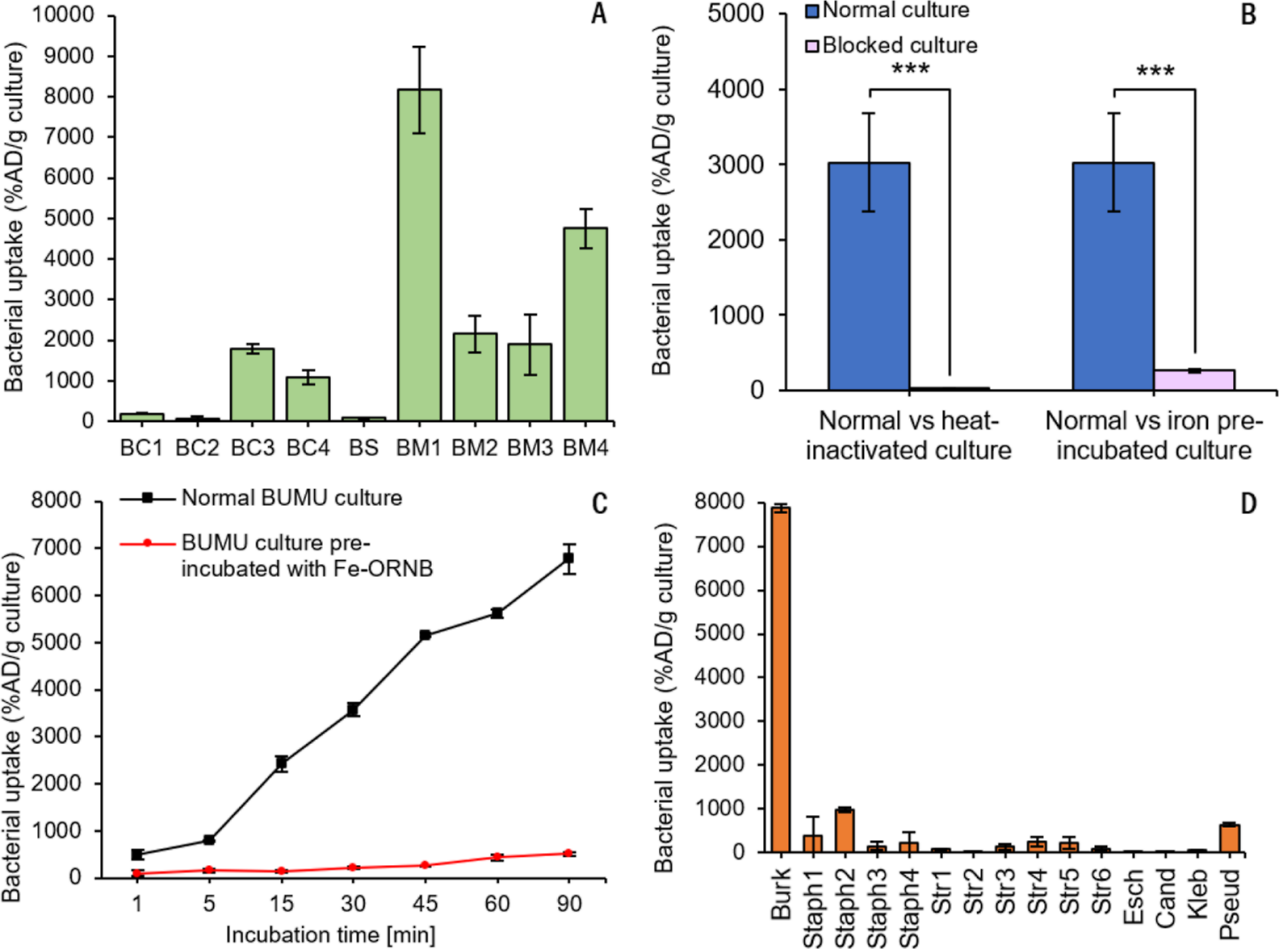
(A) *In vitro* uptake of [^68^Ga]Ga-ORNB after 45 min of incubation in *B. cepacia* complex cultures (BC1 = *B. cenocepacia* 6507, BC2 = *B. cenocepacia* MO 8537, BC3 = *B. cenocepacia* MO 7272, BC4 = *B. cenocepacia* LMG 16656, BS = *B. stabilis* BBB 11382/2014, BM1 = *B. multivorans* AAA 1150/2021, BM2 = *B. multivorans* CF 1865, BM3 = *B. multivorans* CCC 1397/2021, and BM4 = *B. multivorans* 13010). (B) *In vitro* uptake of [^68^Ga]Ga-ORNB after 45 min of incubation in a normal culture of *B. multivorans* LMG 13010 (BUMU) compared to a heat-inactivated culture (90 °C, 20 min) and a culture pre-incubated with iron; ***P < 0.01. (C) *In vitro* uptake of [^68^Ga]Ga-ORNB over time in a normal BUMU culture (black line) and a BUMU culture pre-incubated with Fe-ORNB (red line). (D) *In vitro* uptake of [^68^Ga]Ga-ORNB in different microorganisms compared to uptake in *B. multivorans* after 45 min of incubation (Burk = *B. multivorans*, Staph1 = *Staphylococcus haemolyticus*, Staph2 = *Sta. aureus*, Staph3 = *Sta*. *pseudintermedius*, Staph4 = *Sta. sciuri*, Str1 = *Streptococcus agalactiae*, Str2 = *Str*. *Pyogenes*, Str3 = *Str*. *Constellatus*, Str4 = *Str. Urinalis*, Str5 = *Str*. *Intermedius*, Str6 = *Str*. *Canis*, Esch = *Escherichia Coli*, Cand = *Candida albicans*, Kleb = *Klebsiella pneumoniae*, and Pseud = *Pseudomonas aeruginosa*).

The *in vitro* assays revealed variable uptake of [^68^Ga]Ga-ORNB among different BCC members. The highest uptake was observed in *B. multivorans* 1150A, while cultures of *B. cenocepacia* and *Burkholderia stabilis* showed significantly lower uptake, or even negligible uptake, of [^68^Ga]Ga-ORNB (Figure 2A). Both the heat-inactivated *B. multivorans* LMG 13010 (BUMU) culture and the BUMU culture pre-incubated with FeCl_3_ showed significantly lower [^68^Ga]Ga-ORNB uptake after 45 min of incubation than the normal BUMU culture (Figure 2B). The *in vitro* uptake of [^68^Ga]Ga-ORNB in the normal BUMU culture increased over time and could be blocked by pre-incubation with cold Fe-ORNB (Figure 2C). When [^68^Ga]Ga-ORNB uptake by various respiratory pathogens was compared, the highest uptake was measured in the BUMU culture. No significant uptake was observed for other microbes, with the exception of *Staphylococcus aureus* and *Pseudomonas aeruginosa*, which showed moderate *in vitro* [^68^Ga]Ga-ORNB uptake (Figure 2D).

All of the ^68^Ga-siderophores used to compare *in vitro* uptake in the BUMU culture were radiolabeled with high radiochemical purity (>95%). In addition to [^68^Ga]Ga-ORNB, the BUMU culture showed significant uptake of [^68^Ga]Ga-ferrichrome, [^68^Ga]Ga-ferricrocin, [^68^Ga]Ga-ferrichrysin, and [^68^Ga]Ga-ferrirubin (Figure S4).

### *Ex Vivo* Biodistribution of [^68^Ga]Ga-ORNB in Mice

In non-infected mice, the [^68^Ga]Ga-ORNB complex did not show excessive accumulation in organs and/or tissues of interest and was rapidly excreted through the urinary system (4.79 ± 0.90% ID/g 30 min p.i. versus 2.59 ± 0.31% ID/g 90 min p.i. in the kidneys). The *ex vivo* biodistribution results from normal mice are summarized in Figure S5.

**Figure 3:**
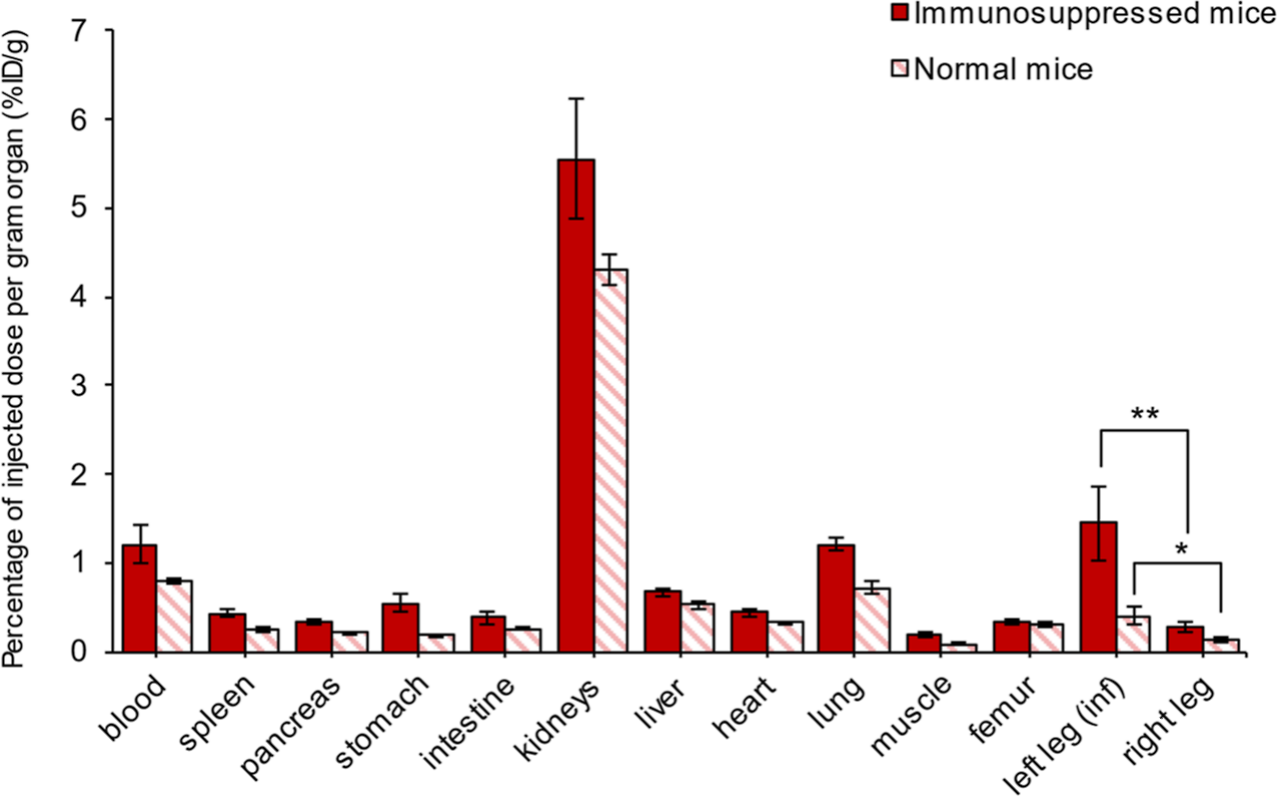
*Ex vivo* biodistribution of [^68^Ga]Ga-ORNB in mice infected with BUMU using the muscle infection model, immunosuppressed and non-immunosuppressed (*n* = 3 per group), 5 h after infection and 45 min after [^68^Ga]Ga-ORNB administration; *P < 0.1; **P < 0.05.

In mice infected with BUMU using the muscle infection model, the complex was found to accumulate more in the infected limb than in the uninfected leg, and this accumulation was significantly enhanced in immunosuppressed mice (1.45 ± 0.41 *vs* 0.28 ± 0.06 in immunosuppressed mice and 0.41 ± 0.01 *vs* 0.14 ± 0.02 in immunocompetent mice). The results from the *ex vivo* biodistribution assay are summarized in Figure 3.

### Animal Imaging Studies

**Figure 4:**
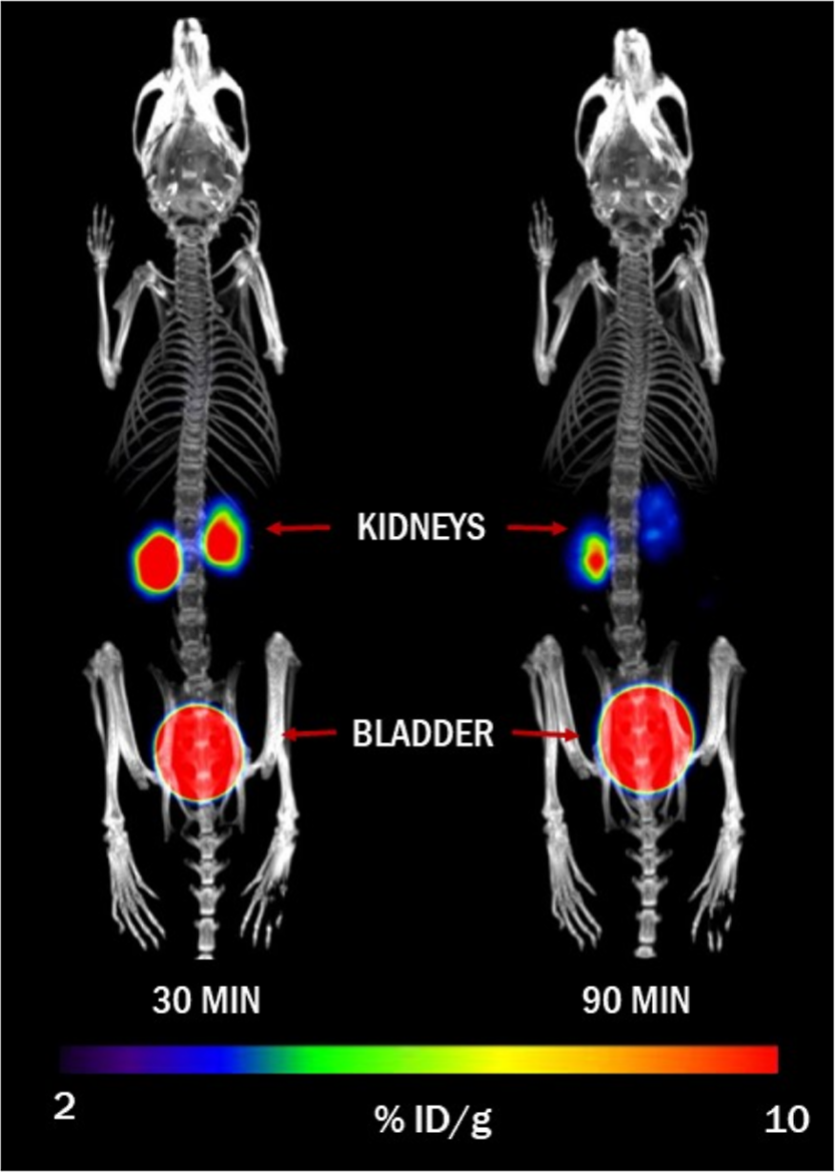
Maximum intensity projection (MIP) PET/CT images of *in vivo* [^68^Ga ]Ga-ORNB biodistribution in normal mice 30 and 90 min after injection of [^68^Ga ]Ga-ORNB.

The results of the *ex vivo* biodistribution assay were confirmed using PET/CT imaging of non-infected mice injected with [^68^Ga]Ga-ORNB. Results from the imaging demonstrated that [^68^Ga]Ga-ORNB was rapidly cleared from the bloodstream, showed no accumulation in major organs and tissues, and was excreted *via* the kidneys (Figure 4).

**Figure 5:**
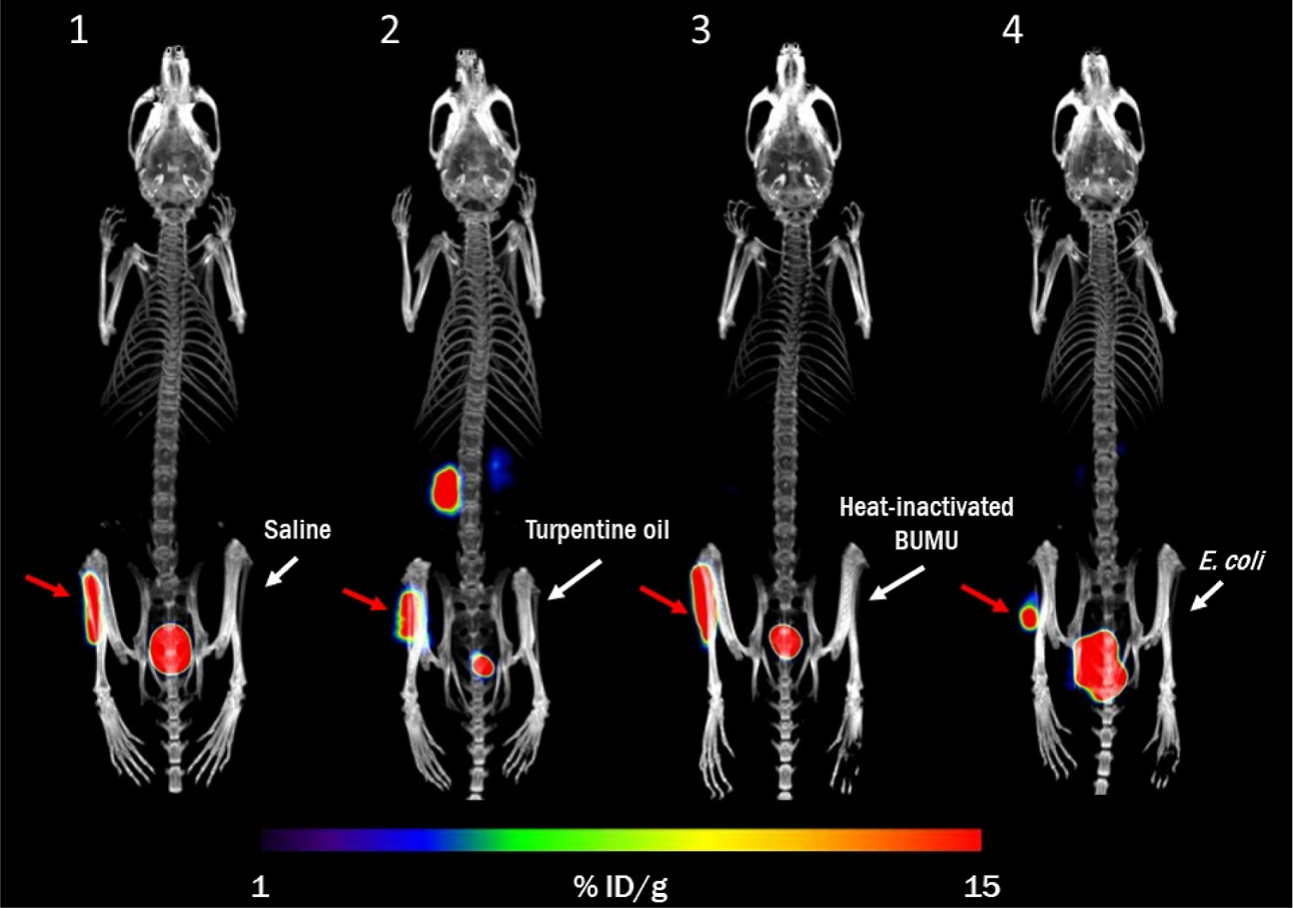
PET/CT *in vivo* imaging of [^68^Ga ]Ga-ORNB biodistribution in a mouse model of BUMU infection in the left hind limb (red arrow) and various agents or microbial cultures in the right hind limb (white arrow): (1) saline, (2) turpentine oil, (3) heat-inactivated BUMU, and (4) *E. coli*. MIP images at 45 min after [^68^Ga ]Ga-ORNB administration.

In a mouse model of BCC myositis, [^68^Ga]Ga-ORNB accumulated in the infected left hind limb but did not accumulate in the control-injected right hind limb. The signal observed in the infected limb was solely due to the underlying bacterial infection since no tracer accumulation was observed in the case of turpentine oil-induced myositis (Figure 5). The radioactive signal in the infected limb tended to decrease with time (Figure S6). In the mouse model of muscle infection, the lowest dose of BUMU detectable by PET imaging was 10^5^ cfu (Figure S7). In the dynamic study, a radioactive signal could be detected in the infected hind limb as early as the first time frame (∼5 min post-injection and post-infection), and signal intensity in the limb increased across subsequent time frames (Figure S8).

**Figure 6:**
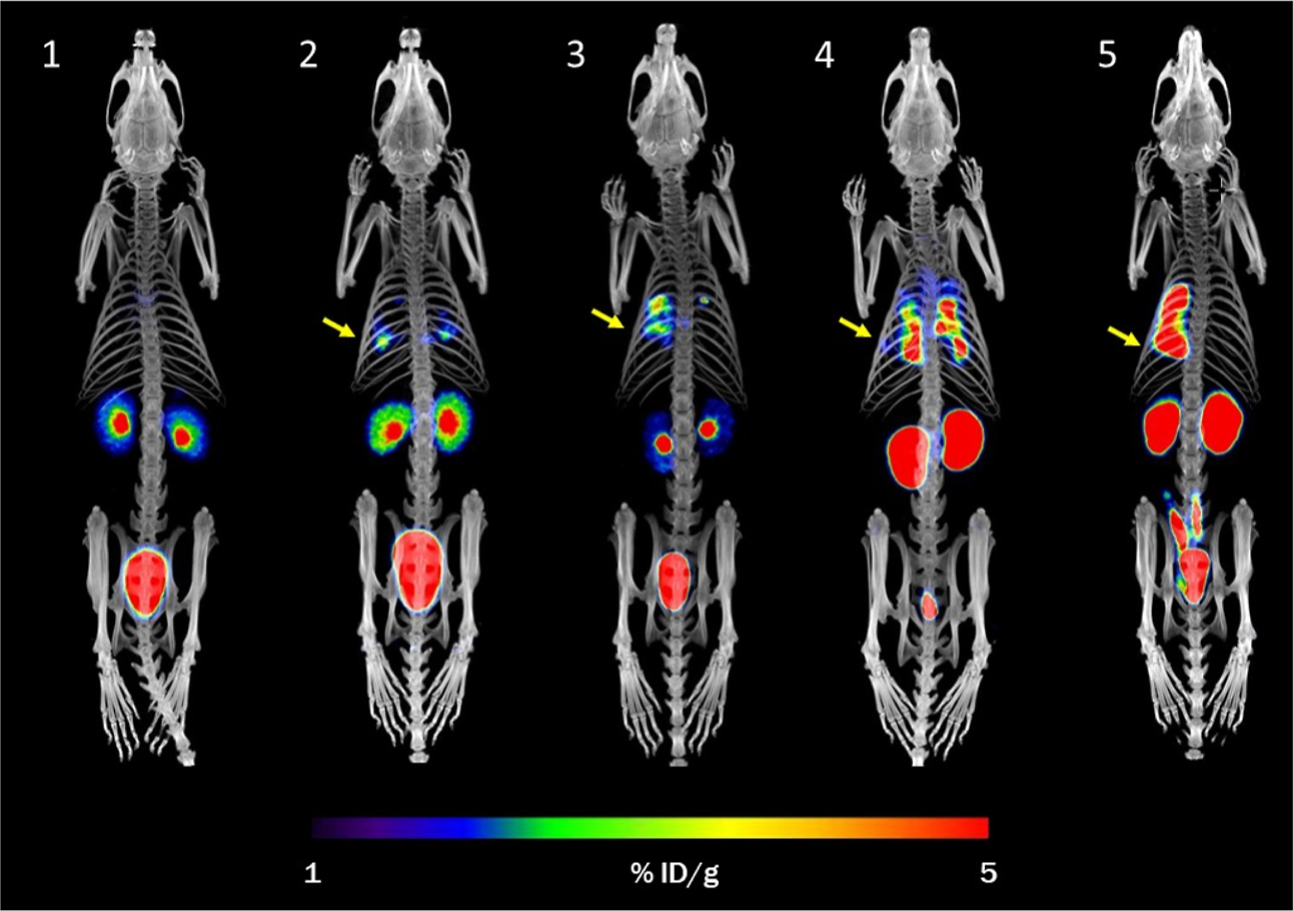
PET/CT MIP images of [^68^Ga ]Ga-ORNB in a control rat (1) and in a rat model of lung infection (BUMU) (2–5) 48–72 h after infection and 45 min after the injection of [^68^Ga ]Ga-ORNB. Yellow arrows indicate the site of infection.

**Figure 7:**
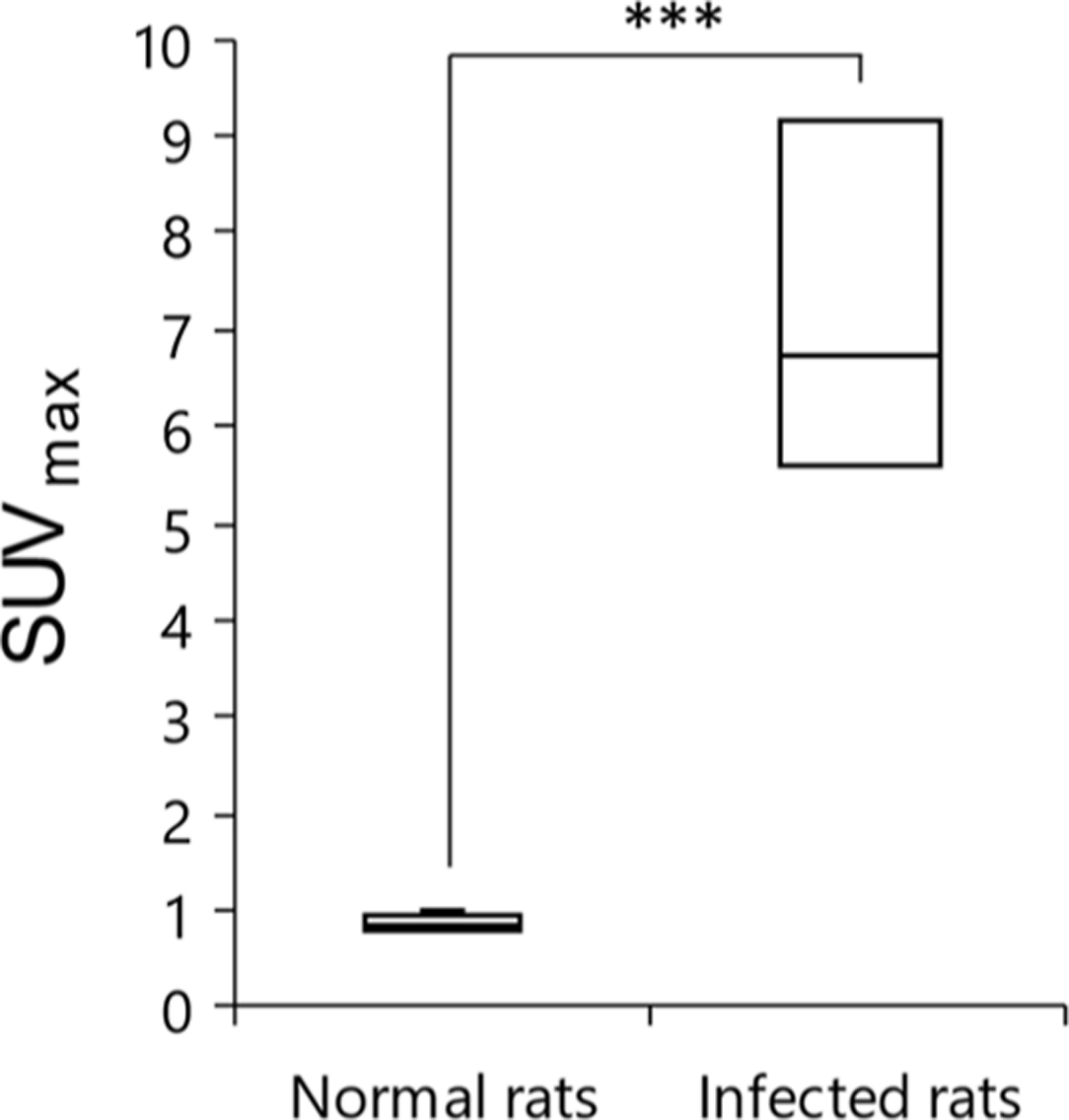
Comparison of radioactive signal uptake in the lungs of non-infected and infected rats (*n* = 4). Results are expressed as the maximal standardized uptake value (SUV_max_); ***P < 0.01.

In a rat model of pulmonary infection, [^68^Ga]Ga-ORNB showed clear accumulation in the lungs of rats infected with BUMU. No radioactive signal was detected in the lungs of non-infected rats (Figure 6). A quantitative analysis revealed significant differences in maximal standardized uptake values (SUV_max_) between non-infected and infected rats (0.88 ± 0.08 versus 7.17 ± 1.48; *P* < 0.01), as displayed in Figure 7.

## Discussion

The accurate diagnosis of bacterial infections in compromised individuals is a challenging task, with the methods currently available for healthcare professionals. The accurate diagnosis of bacterial infections can not only significantly reduce the mortality of infected patients but also ease the pressure to precisely detect the pathogen that is incited by the growing threat of antimicrobial resistance, which discourages the use of empirical antibiotic treatment.^31,32^ The combination of laboratory sampling, microbiological culture, and PCR testing is not always effective for pathogen identification and includes the shortcoming of potentially being unable to distinguish between lower respiratory tract pathogens and upper respiratory tract colonizers.^33^ Although microbiological and/or molecular pathogen detection methods can provide sufficient sensitivity and specificity, they are nevertheless time consuming and cannot localize the site of infection. The most commonly used imaging methods, such as computed tomography or magnetic resonance imaging, rely on structural tissue changes that are non-specific to the infection and appear later during the infectious process.^34^

As this delay may complicate the treatment of infected patients, PET molecular imaging, which depends on functional changes in tissue, appears to be a more appropriate diagnostic tool. However, the radiotracers that are currently used in nuclear medicine to assess infection/inflammation (*e.g.*^18^F-fluordeoxyglucose, ^67^Ga-citrate, ^99m^Tc-methylene diphosphonate, and radiolabeled white blood cells) cannot specifically distinguish between accumulation that is a result of infection versus inflammation.^35^ Moreover, techniques involving leukocyte labeling are often limited by patient immunocompetence.^36^ For these reasons, imaging agents for the detection of specific pathogens have been intensively investigated in recent years. Examples include radiolabeled antimicrobial peptides that result from the host immunity response, various carbohydrate-based substances that are part of microbial metabolism, nucleoside analogs, d-amino acids, para-aminobenzoic acid, antibiotics, antibodies, bacteriophages, aptamers, vitamins, and siderophores.^36−38^ To date, the following gallium-68-labeled siderophores have shown promising results in animal infection models: [^68^Ga]Ga- triacetylfusarinine C for *Aspergillus fumigatus* infection imaging, [^68^Ga]Ga-pyoverdines for *P. aeruginosa* infection imaging, and [^68^Ga]Ga-desferrioxamine B for imaging of various microbial infections.^27−29^ In this study, we investigated the possibility of using the radiolabeled siderophore ornibactin-C6 (Figure 1C) for imaging infections caused by the *B. cepacia* complex. Ferric-ornibactin uptake is mediated by a number of receptors. First, the complex is recognized by the *OrbA* receptor, which is located on the outer membrane and responsible for the uptake of all three conformations of ORNB. Fe-ORNB is then translocated into the periplasmic space in a process that is energetically dependent on the TonB system. Import of the complex into the bacterial cell proceeds *via* periplasmic binding of the protein-dependent ABC complex, which translocates the complex across the inner membrane. Once the complex has been internalized, the process ends with the reduction of iron to ferrous form and release from ORNB.^9,24^ Here, we attempted to evaluate whether replacement of siderophore-bound iron with gallium-68 would influence ORNB uptake by BCC as well as whether [^68^Ga]Ga-ORNB could be useful as a radiotracer for active BCC infection imaging.

We successfully radiolabeled ORNB with gallium-68 with high radiochemical purity. The resulting complex showed low plasma protein binding values and hydrophilic properties and was highly stable in human serum.

According to Ordonez *et al.*, such *in vitro* results represent ideal biochemical properties for a radiotracer.^36^ As expected, that stability of the complex significantly reduced when exposed to solutions containing excess iron or the competitive chelator DTPA. However, the concentrations of both substances in this experiment exceeded clinically relevant levels.^39^ The uptake assays in BCC revealed that *B. multivorans* showed the highest uptake of [^68^Ga]Ga-ORNB, while *B. cenocepacia* and *B. stabilis* showed substantially lower uptake values. These results contradict what was reported in previous studies, more specifically, that both *B. cenocepacia* and *B. stabilis* demonstrate ornibactin production.^9,40^ However, we cannot exclude the induction of genes relevant to ornibactin import systems under *in vivo* conditions, as this was not evaluated in our study. Therefore, the uptake of [^68^Ga]Ga-ORNB by these species deserves further detailed investigation under different conditions using various bacterial strains to assess whether this tracer is applicable to other BCC members. *In vitro* uptake of [^68^Ga]Ga-ORNB in the BUMU culture increased with time and could be blocked by pre-incubating the culture with excess iron-ORNB or heating the culture to 90 °C for 20 min. This confirms that only live bacteria can uptake the siderophore. Various clinically significant respiratory pathogens demonstrated considerably lower uptake of [^68^Ga]Ga-ORNB relative to BUMU. The moderate uptake observed in *P. aeruginosa* was expected as this bacterium is genetically related to BCC.^41^ In addition to [^68^Ga]Ga-ORNB, the BUMU culture also showed uptake of four other ^68^Ga-siderophores, all of which have similar structures: [^68^Ga]Ga-ferrichrome; [^68^Ga]Ga-ferricrocin; [^68^Ga]Ga-ferrichrysin; and [^68^Ga]Ga-ferrirubin. In comparison to [^68^Ga]Ga-ORNB, most of the other ^68^Ga-siderophores have biochemical properties that are not suitable for imaging applications; we have reported these results in a previous study.^26^

Both the *ex vivo* biodistribution study and *in vivo* PET/CT imaging in normal mice provided evidence that [^68^Ga]Ga-ORNB has optimal pharmacokinetic properties. No retention of the complex in the blood or major organs was observed, and the compound was excreted exclusively through the urinary system. Radioactive signal accumulation at the site of infection caused by *B. multivorans* LMG 13010 was detected in both a mouse model of muscle infection and a rat model of lung infection. In the *ex vivo* biodistribution assay, the amount of radioactive signal detected in the hind leg muscles of the infected mouse was greatly affected by immunosuppression. The measured radioactivity, and thus the severity of infection, was significantly higher in the legs of immunosuppressed mice, which is in accordance with the tendency of BCC to infect immunocompromised patients.^42^ The application of PET/CT imaging to the muscle infection model demonstrated that [^68^Ga]Ga-ORNB uptake was specific, as it was possible to distinguish BUMU infection from a sterile inflammation or infection caused by other bacteria. The observed decrease in [^68^Ga]Ga-ORNB accumulation over time at the site of infection was likely due to the fact that muscles are not a suitable environment for the development of BUMU infection due to obligatory aerobic dependence; as such, extrapulmonary BCC infections are uncommon in patients.^43,44^ The lowest infectious dose of BUMU culture that could be imaged with [^68^Ga]Ga-ORNB using PET/CT as early as 5 min post-infection was 10^5^ cfu, which is more than sufficient for the detection of an infection, as bacterial counts in the sputum of CF patients reach values of 10^8^–10^9^ cfu/mL.^45^ The quantification of radioactivity in the lungs of BUMU-infected rats confirmed the significant difference in radioactive signal accumulation between non-infected and infected rats that was observed with *in vivo* PET/CT imaging.

## Conclusions

We have shown that it is possible to label ORNB with gallium-68 both rapidly and with high radiochemical purity. [^68^Ga]Ga-ORNB showed high stability, promising *in vitro* properties, and optimal biodistribution in healthy mice. Moreover, we observed high and specific *in vitro* uptake of [^68^Ga]Ga-ORNB by BCC, a result that was also confirmed through two *in vivo* animal infection models. We believe that [^68^Ga]Ga-ORNB shows strong potential as a perspective PET tracer for *B. cepacia* complex infection imaging. The presented approach has potential clinical applications in disease diagnostics, localization, and monitoring.

## Materials and Methods

### Chemicals, Reagents, and Siderophores

All of the chemicals and reagents used in the study were purchased from commercial sources; they were of analytical grade (purity >95%, as confirmed by HPLC) and used without further purification. Ornibactin-C6, isolated from *Burkholderia vietnamiensis*, and the other siderophores used in this study were purchased from EMC Microcollections GmbH (Tuebingen, Germany), with the exception of Desferal, which was obtained from Novartis (Basel, Switzerland). [^68^Ga]GaCl_3_ was obtained from a ^68^Ga/^68^Ge-generator (Eckert & Ziegler Eurotope GmbH, Berlin, Germany) *via* a fractionated elution method with 0.1 M HCl.^46^

### Radiolabeling of ORNB

The reaction mixture was prepared by adding 5 μg of ORNB dissolved in water (1 μg/μL) to 30 μL of sodium acetate (155 mg/mL in water) and 300 μL of [^68^Ga]GaCl_3_ generator eluate (15–40 MBq). This mixture was incubated for 10 min at room temperature. After incubation, the pH was adjusted to a value between 5 and 6 by the addition of 100 μL of sodium acetate. The radiochemical purity of the final product was analyzed either by reversed-phase high-performance liquid chromatography or by instant thin-layer chromatography, as described below.

### Quality Control of [^68^Ga]Ga-ORNB

The radiochemical purity of [^68^Ga]Ga-ORNB was evaluated by the RP-radioHPLC gradient method (Dionex UltiMate 3000, Thermo Scientific, Waltham, MA, USA) in combination with a radiometric detector (GABI Star, Raytest, Straubenhardt, Germany). A column (Nucleosil 120-5 C18 250 × 40 mm, WATREX, Prague, Czech Republic) with a flow rate of 1 mL/min, oven temperature of 25 °C, and ultraviolet detection at 225 and 250 nm was used with acetonitrile (ACN)/0.1% trifluoroacetic acid (TFA)/H_2_O as the mobile phase with the following gradient: 0–2 min–0% ACN; 2–15 min–0–36% ACN; 15–18 min–36–60% ACN; 18–19.5 min–60% ACN; 19.5–20 min–60–0% ACN; 20–24 min–0% ACN.

Silica-gel-impregnated glass microfiber chromatographic papers (Varian, Lake Forest, CA, USA) were used for radio-iTLC. Chromatographic paper strips containing a sample of the [^68^Ga]Ga-ORNB complex were developed in a chamber saturated with equal parts of ammonium acetate (1 M) and methanol. After development, the stripes were scanned using a radiometric phosphor imager (Cyclone Plus Storage Phosphor System, PerkinElmer, Waltham, MA, USA), and the chromatograms for each strip were evaluated.

### Matrix-Assisted Laser Desorption/Ionization Mass Spectrometry (MALDI MS) and Tandem Mass Spectrometry (MS/MS) of ORNB and ^69/71^Ga-ORNB

Sample preparation: A standard sample of ORNB (EMC Microcollections GmbH, Germany) and ^69/71^Ga-ORNB (^69/71^GaCl_3_:ORNB, 1:1 v/v ratio) were dissolved in 50% methanol to obtain a final concentration of 1 μg/mL. ORNB and ^69/71^Ga-ORNB sample (1 μL) was spotted on the ground steel MALDI target (Bruker Daltonics, Germany) and covered with the α-cyano-4-hydroxycinnemic acid (CHCA) matrix (1 μL, 10 mg/mL in 50% acetonitrile/0.1% trifluoracetic acid).

Samples analysis was performed with the SolariX 12T Fourier-transform ion cyclotron resonance mass spectrometer (Bruker Daltonics, USA) equipped with a Smartbeam II 2 kHz laser (Figures S2 and S3). All data were acquired in positive ion mode and calibrated on the clusters of red phosphorus before analysis. The instrumental parameters were tuned to achieve maximum ion intensities of compounds of interest. Data were collected from *m*/*z* 100 to 1500 with the acquisition of 16 spectra per sample. The time domain file size was set to 2 M. The ion optics were tuned to maximize ion transmission at the defined *m*/*z* range, including collision cell (collision cell voltage: −7.5 V, DC bias: 0.8 V), time-of-flight delay (0.7 ms), and transfer optics (4 MHz, Q1 *m*/*z* 150). The laser was operated at 1000 Hz (50 laser shots/position), and the laser power was tuned at the beginning of the experiment and kept constant for all analyses. Product ion mass spectra were acquired with collision-induced dissociation. The optimum fragmentation spectra were achieved with the collision energy of 15 and 25 V for ORNB (*m*/*z* 709.3724, [M+H]+) and ^69/71^Ga-ORNB (*m*/*z* 775.2721, [M+Ga-2H]+), respectively. Data were processed in DataAnalysis v. 5.2 software (Bruker Daltonics, Germany).

### Stability Tests, Partition Coefficient, and Protein Binding of [^68^Ga]Ga-ORNB

Stability tests were performed by preparing four samples: (1) a 100 μL reaction mixture consisting solely of [^68^Ga]Ga-ORNB; (2) 100 μL of [^68^Ga]Ga-ORNB and 300 μL of human serum; (3) 100 μL of [^68^Ga]Ga-ORNB and 100 μL of diethylenetriaminepentaacetic acid (DTPA, 6 mM); and (4) 100 μL of [^68^Ga]Ga-ORNB and 100 μL of FeCl_3_ (0.1 M). All of the samples were incubated at 37 °C for 30, 60, and 120 min. After incubation, acetonitrile was added to the samples containing human serum; the samples were then further centrifuged (15,000 rpm, 3 min), and the supernatant was analyzed by RP-radioHPLC. Other samples were analyzed directly, either by RP-radioHPLC or by radio-iTLC, as described above.

The partition coefficient (log *P*) was determined by adding 350 μL of the [^68^Ga]Ga-ORNB reaction mixture to 650 μL of phosphate-buffered saline (PBS). A 50 μL sample was taken from this dilution and mixed with 450 μL of PBS and 500 μL of octanol. This solution was stirred on a vortex (1500 rpm, 20 min) and then centrifuged (1 min, 15,000 g) to separate the solvents. A 50 μL sample was collected from both the aqueous and organic phases and then measured on a γ-counter (2480 Wizard^2^ automatic gamma counter; PerkinElmer, Waltham, MA, USA). Log *P* was then calculated based on the measured data (mean of *n* = 6).

Plasma protein binding was assessed by incubating 50 μL of the [^68^Ga]Ga-ORNB reaction mixture with 450 μL of human serum or 450 μL of PBS as a control. Incubation was performed at 37 °C for 30, 60, and 120 min. At each time point, 25 μL of the sample was separated by size-exclusion chromatography (MicroSpin G-50 Columns, Sephadex G-50, GE Healthcare, Buckinghamshire, UK) by centrifugation at 2000*g* for 2 min. Protein binding of [^68^Ga]Ga-ORNB was determined by measuring the distribution of activity between the column (non-protein-bound) and the eluate (protein-bound) using a γ-counter.

### Microbial Strains and Growth Conditions

All of the microbial strains used in this study are listed in Table S1. The bacterial strains required for *in vitro* assays, *ex vivo* biodistribution analyses, and *in vivo* imaging were cultured on Petri dishes containing Columbia blood agar for 24 h at 30 °C. After culturing on solid medium, the bacterial mass was transferred to Erlenmeyer flasks containing 10 mL of Mueller–Hinton broth and shaken at 120 rpm for 24 h at 35 °C. The quantification of bacteria was performed by measuring absorbance at 600 nm using a spectrophotometer (Cary Series UV–vis Spectrophotometer, Agilent Technologies, Santa Clara, USA) and using a standard curve for each bacterial strain to calculate the result.

### *In Vitro* Uptake Assays of [^68^Ga]Ga-ORNB

For the *in vitro* uptake assays, [^68^Ga]Ga-ORNB (*c* ∼ 200 nm) was incubated under various conditions and with different microbial strains for 45 min at 37 °C in Eppendorf tubes shaken at 300 rpm. The incubation was terminated by centrifugation at 15,000 rpm for 5 min, after which the supernatant was removed and the microbial sediment was rinsed with ice-cold Tris buffer (10 mM tris(hydroxymethyl)aminomethane in 0.9% NaCl). The rinsing procedure was repeated twice, after which tubes containing the microbial sediment were weighed and underwent γ-counter measurement. The results were expressed as the percentage of applied dose per gram of microbial culture (% AD/g).

Several assays were performed to investigate the *in vitro* uptake of [^68^Ga]Ga-ORNB by microbial cultures in more detail. To evaluate BCC uptake, [^68^Ga]Ga-ORNB was incubated in various BCC bacteria and handled as described above. (i) To demonstrate the specific and active uptake of [^68^Ga]Ga-ORNB by *Burkholderia*, the BUMU culture was inhibited by heating at 90 °C for 20 min and another BUMU culture was pre-incubated with FeCl_3_, after which the cultures were incubated with [^68^Ga]Ga-ORNB and measured as described above. (ii) To estimate the uptake of [^68^Ga]Ga-ORNB by *Burkholderia* over time and in the presence of an iron–siderophore complex, the BUMU culture was pre-incubated with Fe-ORNB (*c* ∼ 100 μM) for 15 min at 37 °C and 300 rpm. [^68^Ga]Ga-ORNB was then incubated with either a BUMU culture that had been pre-incubated with an iron–siderophore complex or a non-treated BUMU culture for 1, 5, 15, 30, 45, 60, and 90 min, after which the samples were handled as described above. (iii) For comparison of uptake among various respiratory pathogens, [^68^Ga]Ga-ORNB was incubated with different microorganisms as listed in Table S1. The samples were handled as described above. (iv) To compare the uptake of different [^68^Ga]Ga-siderophores by BUMU, desferrioxamine B, desferrioxamine E, ferrichrome, ferrichrome A, triacetylfusarinine C, enterobactin, coprogen, ferricrocin, ferrichrysin, and ferrirubin were radiolabeled with gallium-68. The radiochemical purity of ^68^Ga-siderophores was determined by either RP-radioHPLC or radio-iTLC, as described previously. ^68^Ga-siderophores (*c* ∼ 200 nM) were incubated with BUMU and handled as described above.

### Animal Experiments

Animal experiments were performed on female 8- to 10-week-old Balb/*c* mice and female 8- to 10-week-old Lewis rats (Envigo, Horst, The Netherlands). The animals were acclimatized to laboratory conditions for one week prior to experimental use and housed under standard laboratory conditions on sawdust in individually ventilated cages with free access to animal chow and water. During the experiments, the general health and body weight of the animals were monitored. The number of animals was reduced as much as possible (generally *n* = 3–4 per group and time point) for all *in vivo* experiments. The introduction of bacterial infection into animals, injection, and small animal imaging were all carried out under 2% isoflurane anesthesia (FORANE, Abbott Laboratories, Abbott Park, IL, USA) to minimize animal suffering and prevent animal motion. All of the animal experiments were conducted in accordance with regulations and guidelines of the Czech Animal Protection Act (no. 246/1992) and with the approval of the Czech Ministry of Education, Youth, and Sports (MSMT-9487/2019-5 and MSMT-24421/2021-4) and the Institutional Animal Welfare Committee of the Faculty of Medicine and Dentistry of Palacký University in Olomouc.

### Animal Infection Models

The muscle infection model was performed on immunosuppressed and non-immunosuppressed mice. A group of immunosuppressed mice was injected intraperitoneally (i. p.) with cyclophosphamide (Endoxan, Baxter, Prague, Czech Republic) five and three days prior to infection, as well as on the day of infection (receiving 150, 50, and 50 mg/kg doses, respectively). On the day of infection, all mice were injected intramuscularly (i. m.) with 50 μL of bacterial culture containing BUMU (*c* = 10^4^–10^8^ cfu/mL) into the muscle of the left hind leg. To test the specificity of the *in vivo* uptake of [^68^Ga]Ga-ORNB, 50 μL of different bacterial cultures (BUMU or *E. coli*; live or heat-inactivated), turpentine oil (to induce sterile inflammation), or saline solution were injected into the right hind leg muscle. The microbial infections were allowed to develop for 5 min for the dynamic imaging study, for 45 min–48 h for the monitoring of [^68^Ga]Ga-ORNB uptake, and for 5 h for *ex vivo* biodistribution studies and *in vivo* specificity testing. The induction of sterile inflammation lasted 24 h.

For the lung infection model, immunosuppressed rats were treated with cyclophosphamide (Endoxan, Baxter, Prague, Czech Republic) five days and one day before infection (75 mg/kg, i. p.). Rats were infected intratracheally with 100 μL of BUMU culture (*c* = 7–8 × 10^8^ cfu/mL) under inhalation anesthesia. BUMU was administered using the TELE PACK VET X LED system equipped with a rigid endoscope (Karl Storz GmbH & Co. KG, Tuttlingen, Germany). Rats underwent PET/CT imaging 5–72 h after inoculation.

### *Ex Vivo* Biodistribution in Mice

The biodistribution studies were performed on non-infected and immunosuppressed or non-immunosuppressed infected mice. Immunosuppression in mice was induced as described above. Mice were injected retro-orbitally (r.o.) with [^68^Ga]Ga-ORNB (1–2 MBq, approximately ∼0.5 μg of ORNB). All mice were sacrificed under general anesthesia by cervical dislocation followed by exsanguination. Non-infected mice were sacrificed 30 and 90 min after injection, while infected mice were sacrificed 45 min after injection. The blood, spleen, pancreas, stomach, intestines, kidneys, liver, heart, lungs, muscle, and bone were collected; subsequently, the organs and tissues were weighed, and radioactivity was measured using a γ-counter. The biodistribution data were calculated as the percentage of injected dose per gram of tissue (% ID/g).

### Animal Imaging Studies

Experimental animals under isoflurane anesthesia were r.o. injected with [^68^Ga]Ga-ORNB (approximately ∼0.5 μg of ORNB) at a dose of 2–7 MBq per animal and placed in a dorsoventral position in the Mediso NanoScan PET/CT imaging system for small animals (Mediso Medical Imaging Systems, Budapest, Hungary). After the administration of [^68^Ga]Ga-ORNB, static imaging was initiated 30 and 90 min p.i. for non-infection imaging studies and 45 min p.i. for infection imaging studies. Dynamic imaging studies were started ∼5 min p.i. Single FOV PET scans (98.5 mm) for mice and double FOV PET scans (2 × 98.5 mm) for rats were performed, followed by whole body helical CT scan (50 kVp/980 μA, 720 projections). Image reconstruction was performed *via* Mediso Tera-Tomo 3D PET iterative reconstruction (Mediso Medical Imaging Systems, Budapest, Hungary). The images were visualized, processed, and quantified in the Mediso InterView FUSION (Mediso Medical Imaging Systems, Budapest, Hungary). Quantitative analyses were performed on images of non-infected rats and rats with lung infections. The images were normalized to injected activity and animal weight. The results were expressed as percentage of injected dose per gram tissue (% ID/g).

### Statistical and Data Analyses

All of the statistical analyses were performed using Microsoft Office 365 Excel (Microsoft Corporation, Redmond, WA, USA). Data were analyzed using an unpaired two-tailed Student’s *t*-test. All of the presented graphs include error bars, which denote the standard deviation. Other data, including the *in vitro* characterization of [^68^Ga]Ga-ORNB, are reported as the mean value ± standard deviation.

## Supporting Information Available

Quality control of [^68^Ga]Ga-ORNB; high-resolution MS spectra of an ORNB standard sample and a 69/71Ga-ORNB sample; product ion scan from *m*/*z* 709.3724 and *m*/*z* 775.2721 acquired by MALDI MS/MS; *in vitro* uptake of ^68^Ga-labeled siderophores in BUMU; *ex vivo* biodistribution assay of [^68^Ga]Ga-ORNB in normal mice; PET/CT *in vivo* imaging of [^68^Ga]Ga-ORNB biodistribution in the BUMU muscle infection model at various time points and with various infectious doses; PET *in vivo* dynamic study of [^68^Ga]Ga-ORNB biodistribution in the BUMU muscle infection model; and list of microbial strains used in the study (PDF)
Molecular formula strings, SMILES (CSV)

## Abbreviations Used

ACN: acetonitrile
BCC: *Burkholderia cepacia* complex
BUMU: *Bukrholderia multivorans* LMG 130 10
CF: cystic fibrosis
DTPA: diethylenetriaminepentaacetic acid
radio-iTLC: radio instant thin layer chromatography
MALDI MS: matrix-assisted laser desorption/ionization mass spectrometry
MIP: maximum intensity projection
MLST: multi-locus sequence typing
MS/MS: tandem mass spectrometry
ORNB: ornibactin
PBS: phosphate-buffered saline
PCR: polymerase chain reaction
PET: positron emission tomography
RP-radioHPLC: reversed-phase radio high performance liquid chromatography
SUV_max_: maximal standardized uptake value
TFA: trifluoroacetic acid
