# Supplementary material for: [68Ga]Ga-Ornibactin for Burkholderia cepacia complex Infection Imaging Using Positron Emission Tomography
Source: J Med Chem. 2023 May 30;66(11):7584–93. doi: 10.1021/acs.jmedchem.3c00469 (PMC10258796; doi:10.1021/acs.jmedchem.3c00469)
Supplement: Supplementary file 1 — jm3c00469_si_001.pdf [file jm3c00469_si_001.pdf]

SUPPORTING INFORMATION

[^{68}Ga]Ga-Ornibactin for *Burkholderia cepacia* complex Infection Imaging Using Positron Emission Tomography

Katerina Bendova[1], Vladislav Raclavsky[2], Radko Novotny[2], Dominika Luptakova[3], Miroslav Popper[1], Zbynek Novy[1], Marian Hajduch[1,4]*, and Milos Petrik[1]*

1 Institute of Molecular and Translational Medicine, Faculty of Medicine and Dentistry and Czech Advanced Technology and Research Institute, Palacky University, Olomouc 779 00, Czech Republic

2 Department of Microbiology, Faculty of Medicine and Dentistry, Palacky University and University Hospital, Olomouc 775 15, Czech Republic

3 Institute of Microbiology of the Czech Academy of Sciences, Laboratory of Molecular Structure Characterization, Prague 4 142 20, Czech Republic

4 Laboratory of Experimental Medicine, University Hospital, Olomouc 779 00, Czech Republic

***Correspondence:** milos.petrik@upol.cz, marian.hajduch@upol.cz

TABLE OF CONTENTS

1. Quality control of [^{68}Ga]Ga-ORNB S2
2. High-resolution MS spectra of an ORNB standard sample and a $^{69/71}$Ga-ORNB sample S5
3. Product ion scan from *m/z* 709.3724 and *m/z* 775.2721 acquired by MALDI MS/MS S5
4. *In vitro* uptake of ^{68}Ga-labeled siderophores in BUMU S6
5. Biodistribution assays of [^{68}Ga]Ga-ORNB in mice models
   5.1. *Ex vivo* biodistribution assay in normal mice S6
   5.2. PET/CT *in vivo* imaging in the BUMU muscle infection model at various timepoints S7
   5.3. PET/CT *in vivo* imaging in the BUMU muscle infection model with various inf. doses S8
   5.4. PET *in vivo* dynamic study in the BUMU muscle infection model S9
6. List of microbial strains used in the study S10

Figure S1: Quality control of [^{68}Ga]Ga-ORNB performed on **(A)** RP-radioHPLC and **(B)** radio-iTLC-SG. **(C)** RP-HPLC UV trace of cold Ga-ORNB (λ = 250 nm). **(D)** RP-HPLC UV trace (pink) and radiodetector trace (black) of [^{68}Ga]Ga-ORNB with co-injection with cold Ga-ORNB (λ = 250 nm). **(E)** RP-HPLC UV trace of [^{68}Ga]Ga-ORNB. **(F)** RP-HPLC UV trace of ORNB.

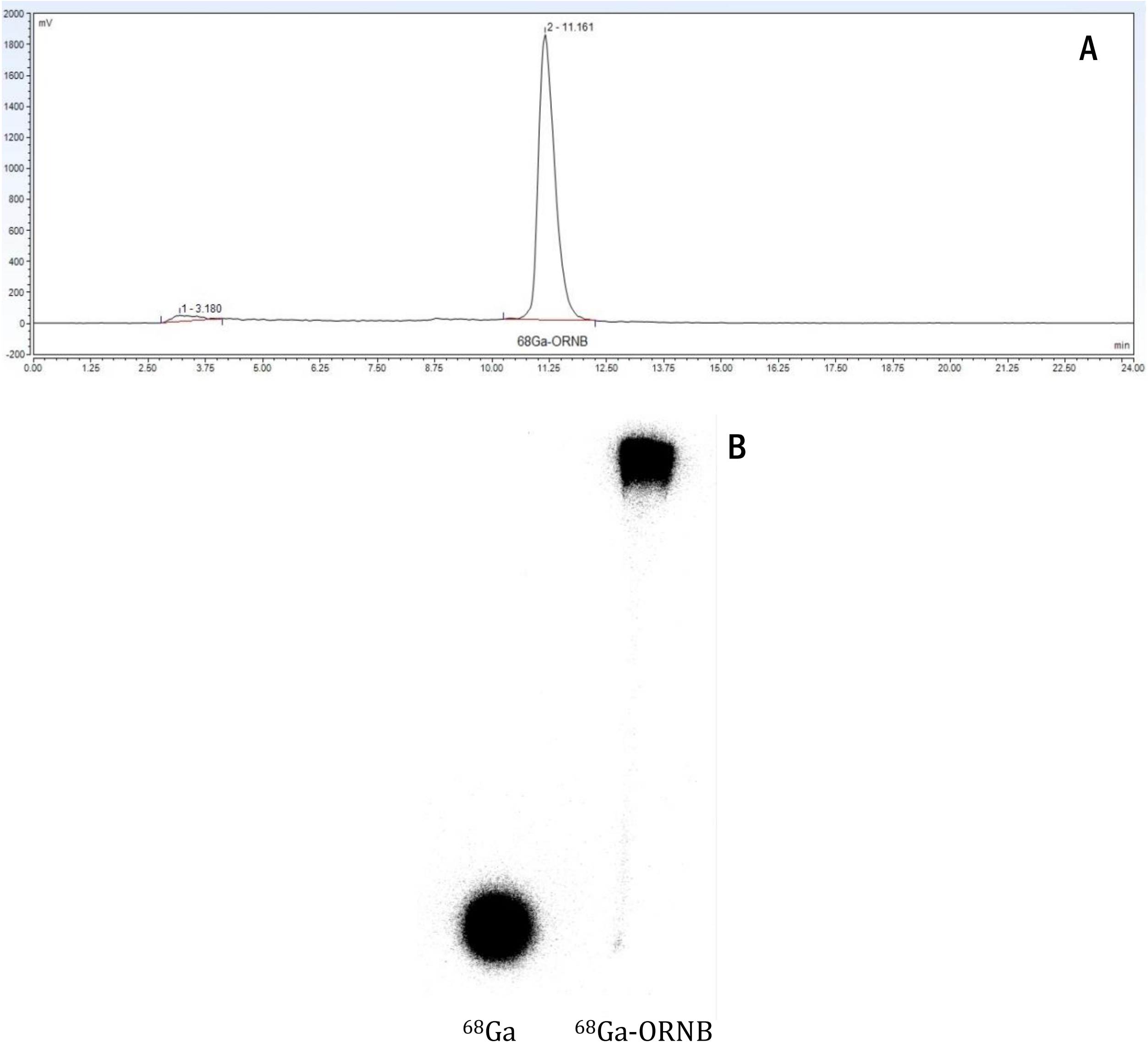

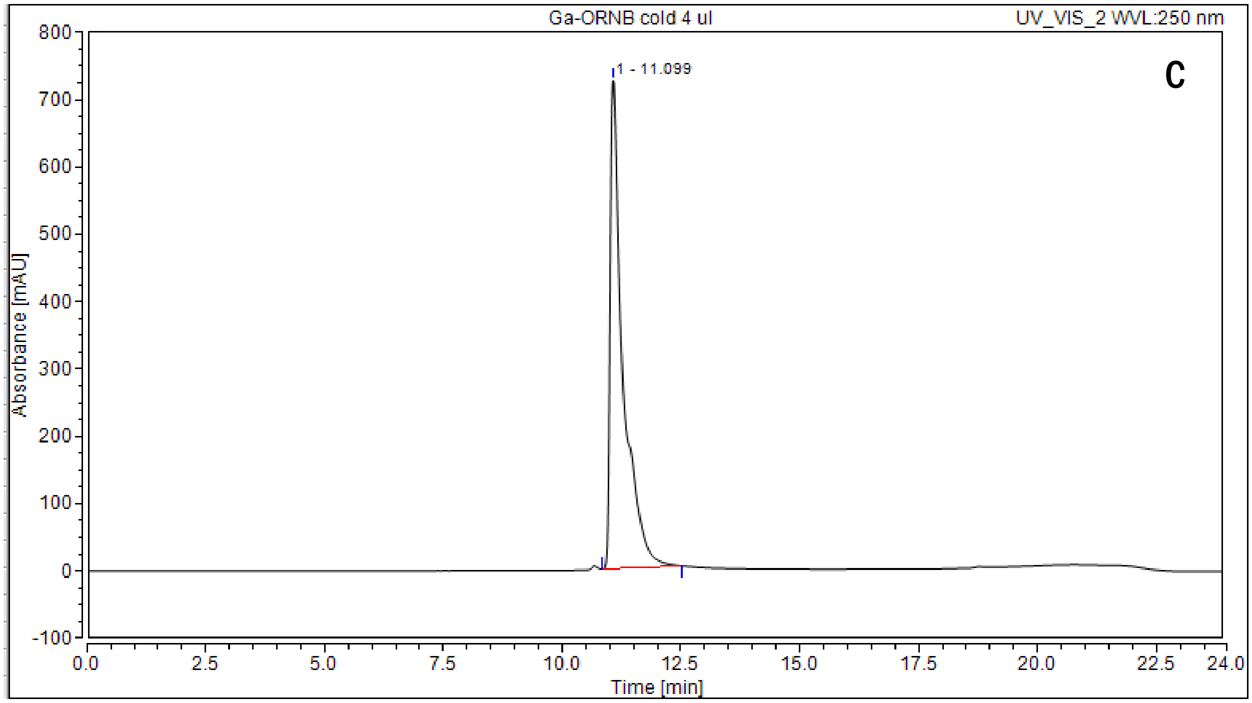
Ga-ORNB cold 4 ul
UV_VIS_2 WVL:250 nm
C
1 - 11.099
Absorbance [mAU]
Time [min]

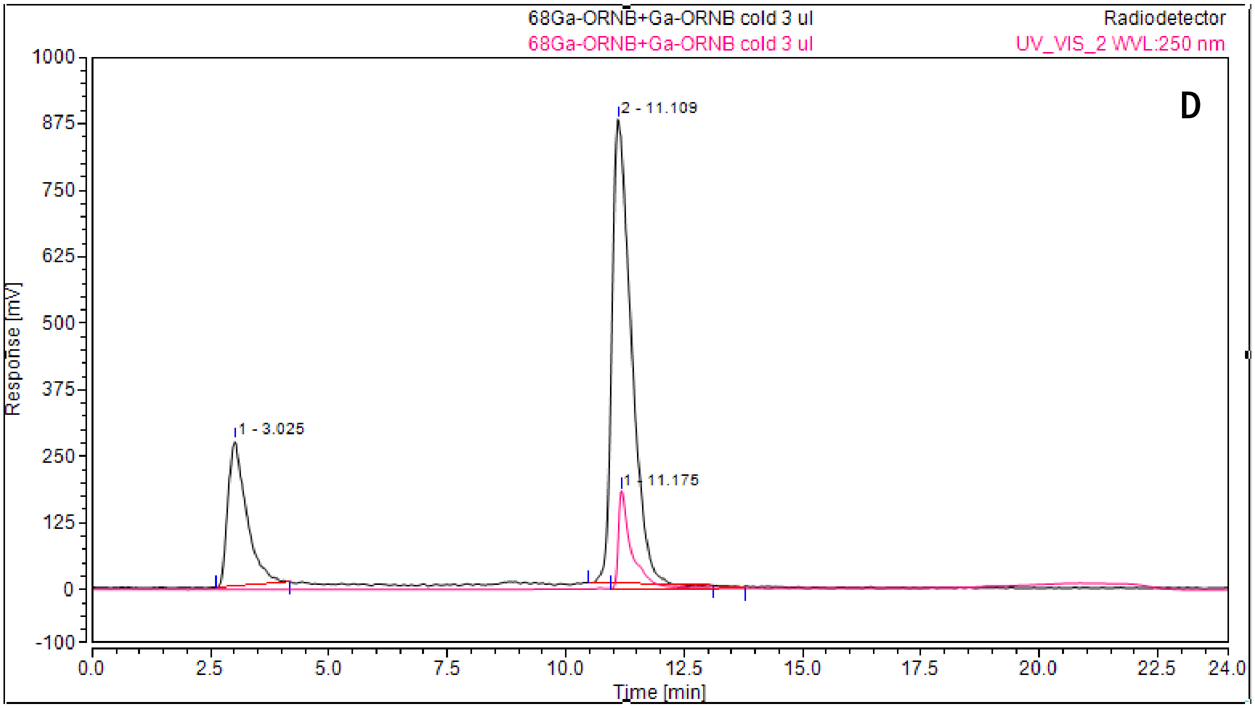
68Ga-ORNB+Ga-ORNB cold 3 ul
Radiodetector
68Ga-ORNB+Ga-ORNB cold 3 ul
UV_VIS_2 WVL:250 nm
D
2 - 11.109
1 - 3.025
1 - 11.175
Response [mV]
Time [min]

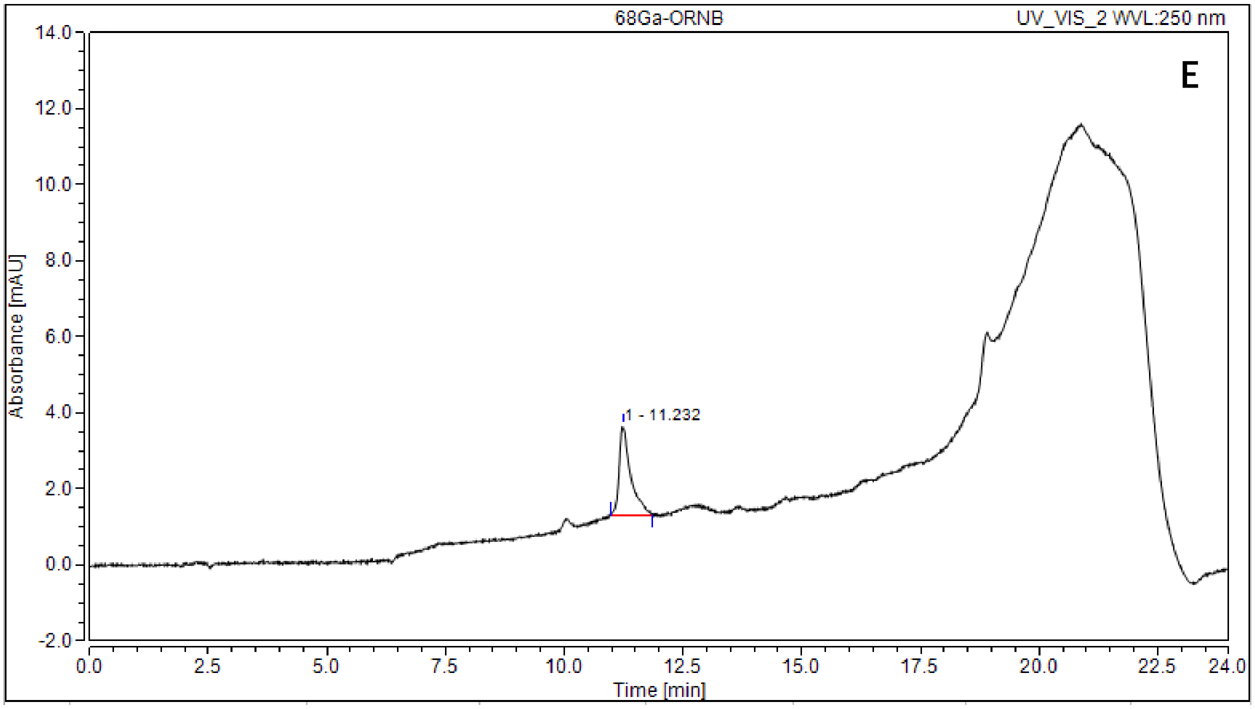
68Ga-ORNB
UV_VIS_2 WVL:250 nm
E
1 - 11.232
Absorbance [mAU]
Time [min]
14.0
12.0
10.0
8.0
6.0
4.0
2.0
0.0
-2.0
0.0
2.5
5.0
7.5
10.0
12.5
15.0
17.5
20.0
22.5
24.0

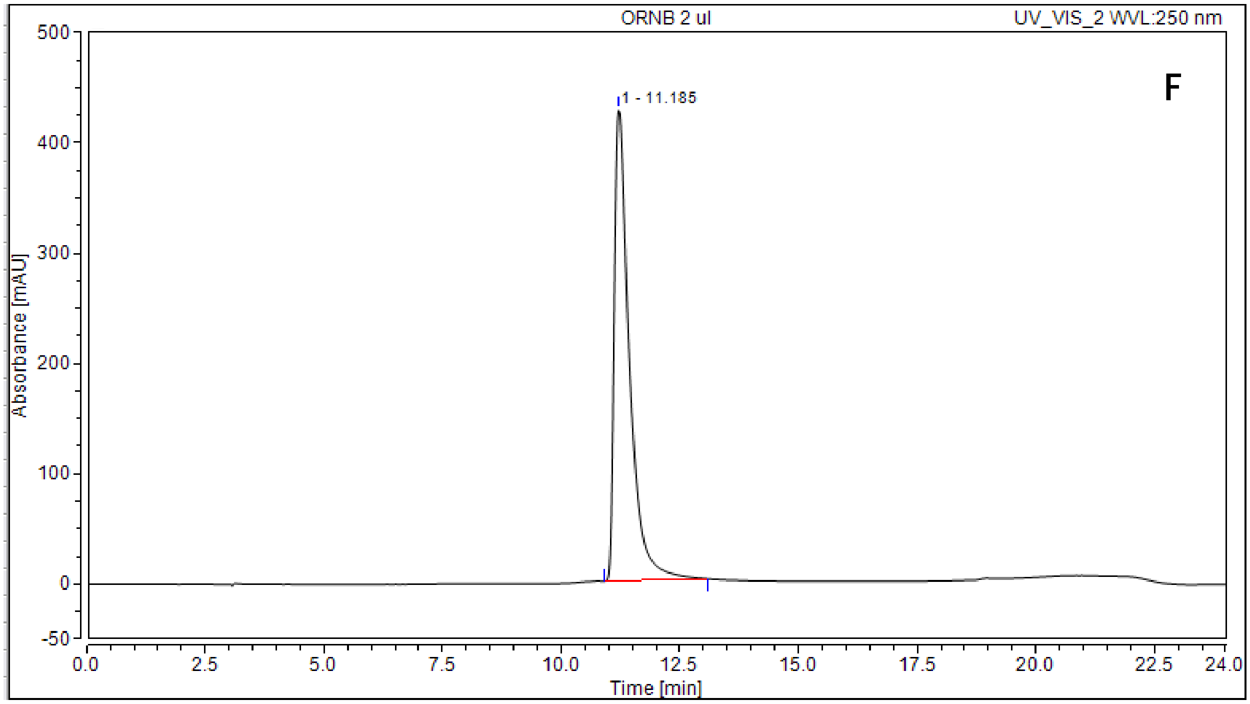
ORNB 2 ul
UV_VIS_2 WVL:250 nm
F
1 - 11.185
Absorbance [mAU]
Time [min]
500
400
300
200
100
0
-50
0.0
2.5
5.0
7.5
10.0
12.5
15.0
17.5
20.0
22.5
24.0

Figure S2: High-resolution MS spectra of **(A)** an ORNB standard sample and **(B)** a $^{69/71}$Ga-ORNB sample acquired by matrix-assisted laser desorption/ionization (MALDI) MS in the 100 – 1500 *m/z* mass range. Zoom into the molecular mass region on the conventional mass spectrum showing the detection of **(C)** protonated, sodiated, and pottasiated molecular adducts of ORNB and **(D)** a characteristic isotopic profile of the protonated molecular adduct of $^{69/71}$Ga-ORNB. CHCA, α-cyano-4-hydroxycinnemic acid; calc., calculated mass.

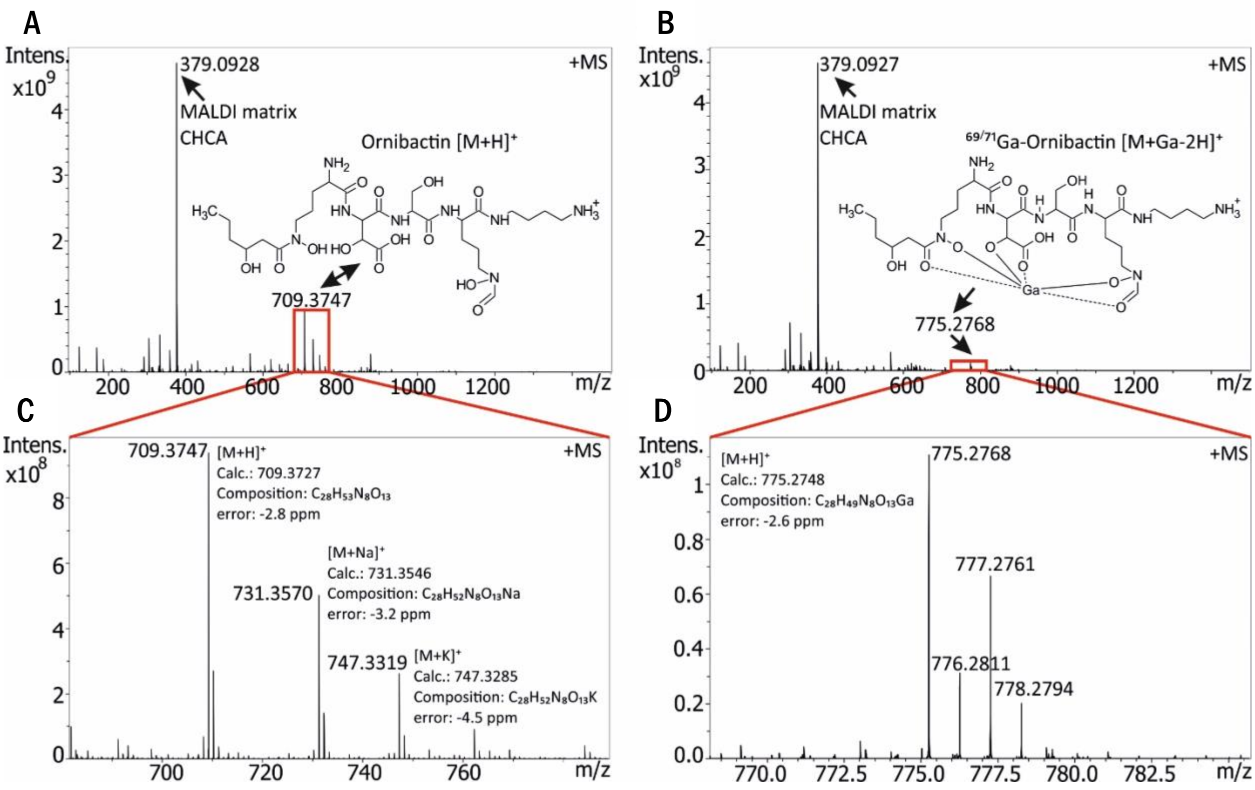

Figure S3: Product ion scan: from **(A)** *m/z* 709.3724 (ORNB, [M+H]+; and **(B)** *m/z* 775.2721 ($^{69/71}$Ga-ORNB, [M+Ga-2H]+; acquired by MALDI MS/MS with the 15 V and 25 V collision energy, respectively.

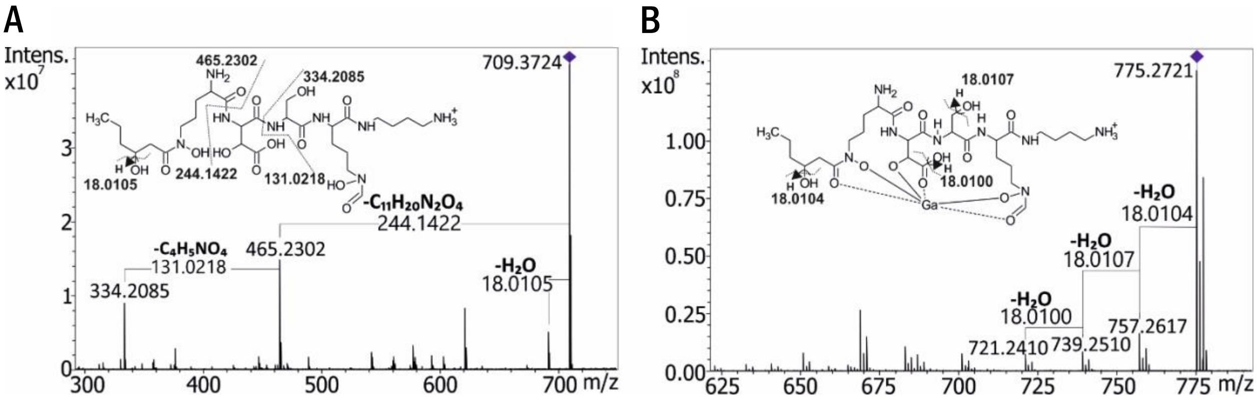

Figure S4: *In vitro* uptake of various ^{68}Ga-labeled siderophores in *Burkholderia multivorans* LMG 13010 after 45 min of incubation. (ORNB = [^{68}Ga]Ga-Ornibactin, ENT = [^{68}Ga]Ga-Enterobactin, TAFC = [^{68}Ga]Ga-Triacetylfusarinin C, COP = [^{68}Ga]Ga-Coprogen, FCH = [^{68}Ga]Ga-Ferrichrome, FCH A = [^{68}Ga]Ga-Ferrichrome A, DFO = [^{68}Ga]Ga-Desferrioxamine, FCR = [^{68}Ga]Ga-Ferricrocin, FCHR = [^{68}Ga]Ga-Ferrichrysin, FRU = [^{68}Ga]Ga-Ferrirubin, FOX E = [^{68}Ga]Ga-Ferrioxamine E)

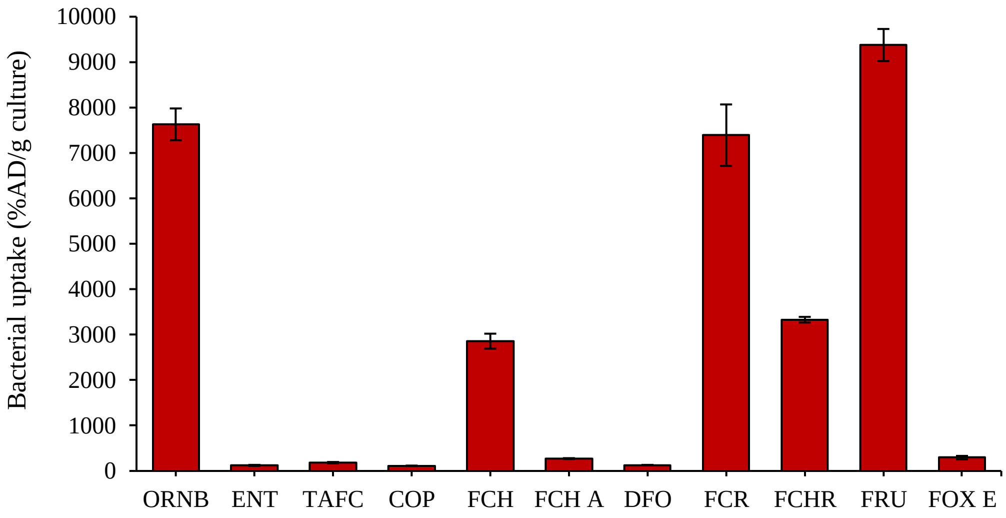

Figure S5: *Ex vivo* biodistribution assay of [^{68}Ga]Ga-ORNB in normal mice 30 and 90 min after [^{68}Ga]Ga-ORNB administration.

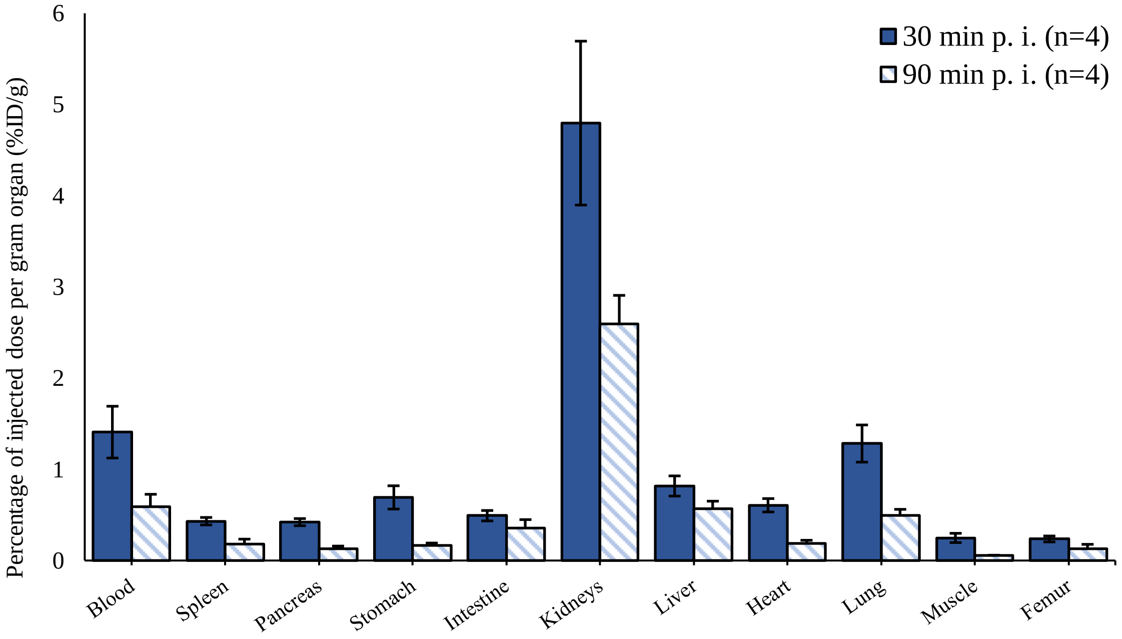

Figure S6: PET/CT *in vivo* imaging of [^{68}Ga]Ga-ORNB biodistribution in the BUMU muscle infection model in mice at 5, 24 and 48 h after infection and 45 min after [^{68}Ga]Ga-ORNB administration (maximum intensity projections images). Red arrow indicates the infection.

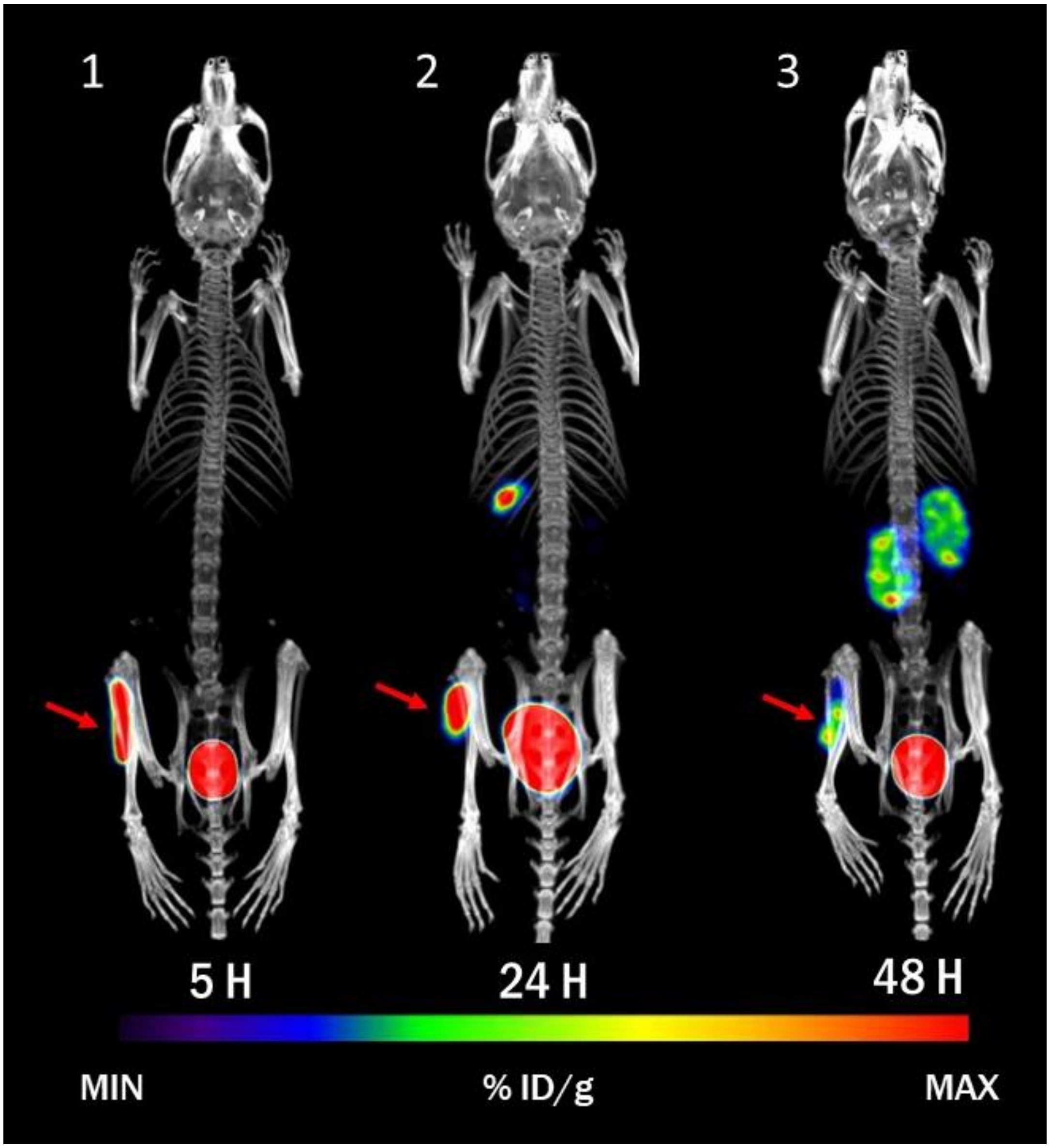

Figure S7: PET/CT *in vivo* imaging of [^{68}Ga]Ga-ORNB biodistribution in the BUMU muscle infection model with various infectious doses in mice 5 h after infection and 45 min after [^{68}Ga]Ga-ORNB administration (maximum intensity projections images). Yellow arrow indicates the infection.

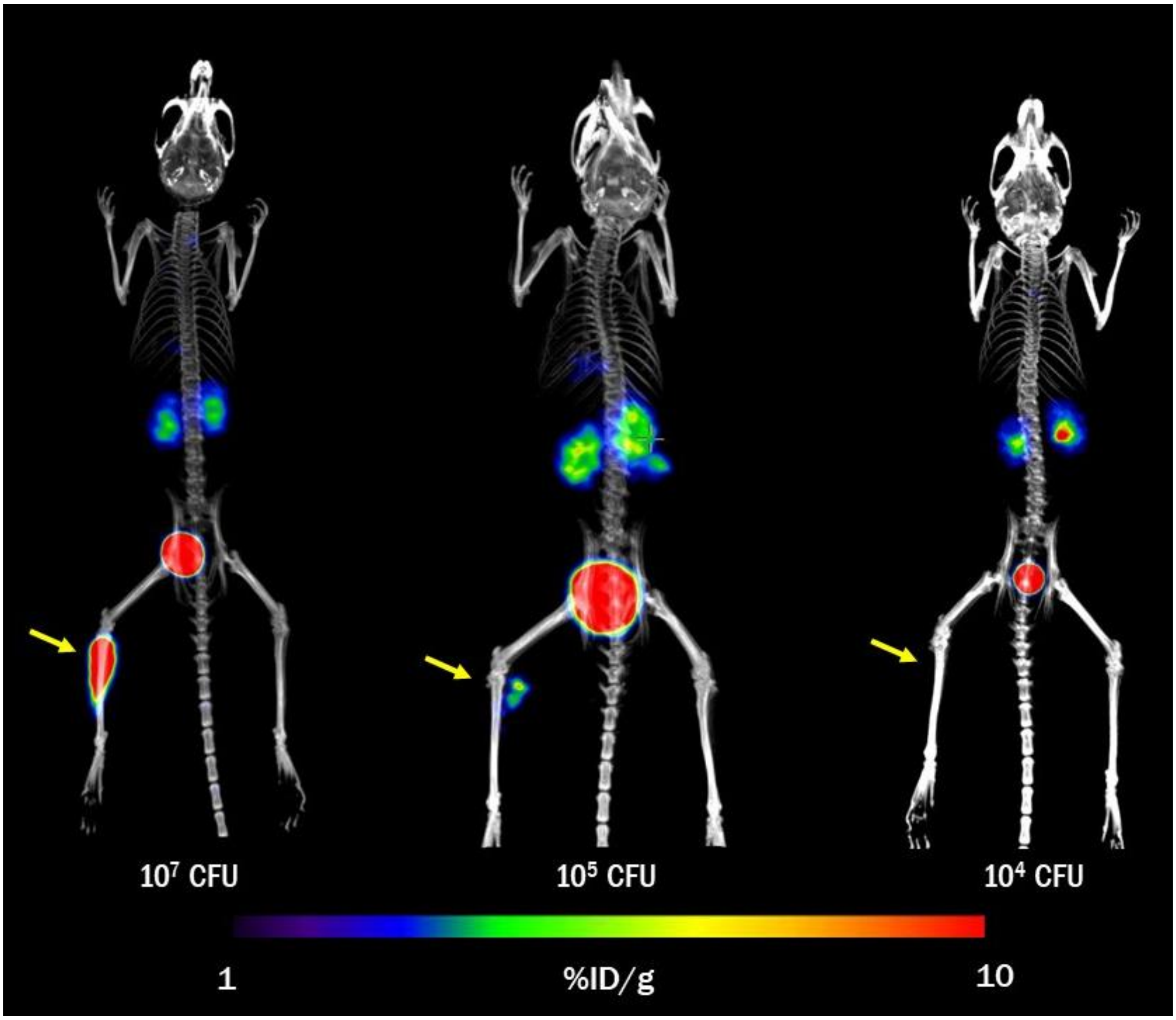

Figure S8: PET *in vivo* dynamic study of [^{68}Ga]Ga-ORNB biodistribution in the BUMU muscle infection model 5-90 min after infection and [^{68}Ga]Ga-ORNB administration (maximum intensity projections images). H = heart, K = kidneys, B = bladder, **INF** = site of infection.

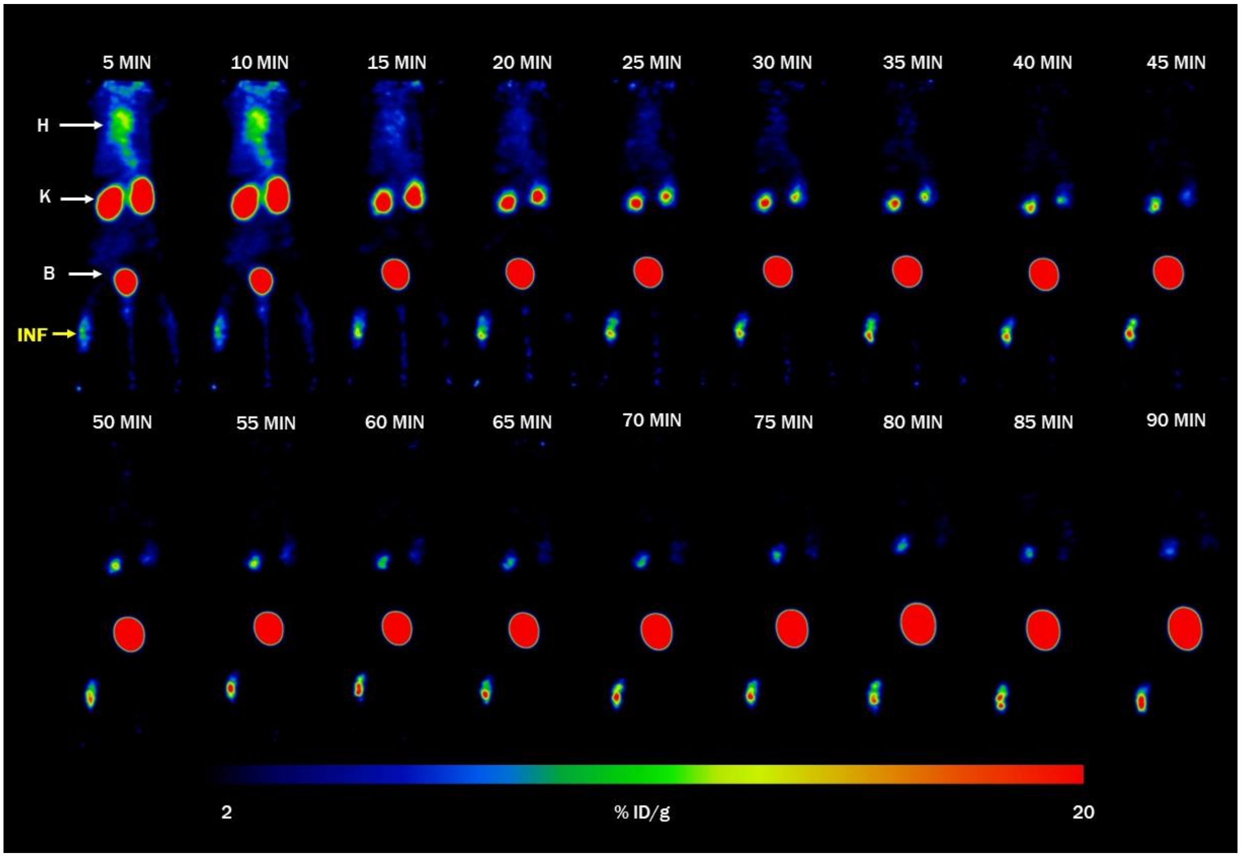

Table S1: List of microbial strains used in the study. The list is composed of two parts: **(A)** list of microbes purchased from commercial culture collections and **(B)** list of microbes obtained from the collection of Department of Microbiology, Faculty of Medicine and Dentistry, Palacky University Olomouc.

<table>
<tr><th colspan="3">(A) CULTURE COLLECTIONS</th></tr>
<tr><th>Species</th><th colspan="2">Origin and strain number</th></tr>
<tr><td>Burkholderia cenocepacia</td><td colspan="2">LMG 16656</td></tr>
<tr><td>Burkholderia multivorans</td><td colspan="2">LMG 13010</td></tr>
<tr><td>Candida albicans</td><td colspan="2">ATCC 90028</td></tr>
<tr><td>Escherichia coli</td><td colspan="2">ATCC 25922</td></tr>
<tr><td>Klebsiella pneumoniae</td><td colspan="2">NCTC 13442</td></tr>
<tr><td>Pseudomonas aeruginosa</td><td colspan="2">ATCC 15692</td></tr>
<tr><th colspan="3">(B) PALACKÝ UNIVERSITY – DEPARTMENT OF MICROBIOLOGY, ISOLATES FROM CLINICAL SAMPLES</th></tr>
<tr><th>Species</th><th>Isolate number (MLST type)</th><th>Origin</th></tr>
<tr><td>Burkholderia cenocepacia</td><td>CF 6507 (= ST-761)</td><td>Nasopharyngeal swab in a cystic fibrosis patient, 27-y old woman, chronic infection</td></tr>
<tr><td>Burkholderia cenocepacia</td><td>*MO 7272 (= ST-32)</td><td>Cystic fibrosis patient</td></tr>
<tr><td>Burkholderia cenocepacia</td><td>*MO 8537 (= ST-234)</td><td>Cystic fibrosis patient</td></tr>
<tr><td>Burkholderia multivorans</td><td>CCC 1397/2021</td><td>Bronchial secretion, Covid-19 patient, 22-y old man, respiratory failure</td></tr>
<tr><td>Burkholderia multivorans</td><td>AAA 1150/2021</td><td>Nasopharyngeal swab, Covid-19 patient, 85-y old man, viral pneumonia</td></tr>
<tr><td>Burkholderia multivorans</td><td>CF 1865 (= ST-439)</td><td>Wound, oncology patient, 68-y old woman, malignant neoplasm of mouth</td></tr>
<tr><td>Burkholderia stabilis</td><td>BBB 11382/2014</td><td>Vaginal swab, pregnancy, 35-y old woman, threatened abortion</td></tr>
<tr><td>Staphylococcus aureus</td><td>CF 184</td><td>Sputum, cystic fibrosis patient, 24-y old man, chronic infection</td></tr>
<tr><td>Staphylococcus haemolyticus</td><td>CF 1680</td><td>Rectal swab, surgery patient, 54-y old woman, fourth degree hemorrhoids</td></tr>
<tr><td>Staphylococcus pseudintermedius</td><td>CF 3460</td><td>Healthy dog</td></tr>
<tr><td>Staphylococcus sciuri</td><td>CF 3462</td><td>Wound, trauma, 70-y old man</td></tr>
<tr><td>Streptococcus agalactiae</td><td>CF 2276</td><td>Rectal swab, surgery patient, 75-y old woman, malignant neoplasm of ascending colon</td></tr>
<tr><td>Streptococcus canis</td><td>CF 6893 (CF 36/2021)</td><td>Nasopharyngeal swab, 7-old girl, selective defficiency of IgG classes</td></tr>
<tr><td>Streptococcus constellatus</td><td>CF 6732</td><td>Mouth wash, oncology patient, 65-y old man, non-small cell lung cancer</td></tr>
</table>

Streptococcus intermedius	CF 6734	Sputum, cystic fibrosis patient, 11-y old boy, stable disease
Streptococcus pyogenes	CF 361	Nasopharygeal swab, cystic fibrosis patient, 7-y old boy, tonsilitis
Streptococcus urinalis	CF 2778	Skin lesion swab, 35-y old man, balanitis

*Isolates marked MO were kindly gifted by Dr. Pavel Dřevínek, Department of Microbiology, Charles University Prague
